# Supplementary material for: Benchmark problems for dynamic modeling of intracellular processes
Source: Bioinformatics. 2019 Jan 8;35(17):3073–82. doi: 10.1093/bioinformatics/btz020 (PMC6735869; doi:10.1093/bioinformatics/btz020)
Supplement: btz020_Supplementary_Information [file btz020_supplementary_information.pdf]

Supplementary Information

Benchmark Problems for Dynamic Modeling of Intracellular Processes

Helge Hass [1,2†], Carolin Loos [3,4†], Elba Raimundez Alvarez [3,4], Jens Timmer [1,2,5], Jan Hasenauer [3,4*], and Clemens Kreutz [1,2*]

[1]Center for Systems Biology (ZBSA), University of Freiburg, 79104 Freiburg, Germany

[2]Center for Data Analysis and Modelling (FDM), University of Freiburg, 79104 Freiburg, Germany

[3]Helmholtz Zentrum München - German Research Center for Environmental Health, Institute of Computational Biology, Neuherberg 85764, Germany

[4]Technische Universität München, Center for Mathematics, Chair of Mathematical Modeling of Biological Systems, Garching 85748, Germany

[5]BIOSS Centre for Biological Signalling Studies, University of Freiburg, 79104 Freiburg, Germany

[†]These authors contributed equally.

[*]To whom correspondence should be addressed.

Contents

1 Detailed description of provided files **3**

1.1 General overview . . . 3

1.2 Condition-specific model files . . . 5

1.3 Condition-specific data files . . . 6

2 Usage of models and optional simulation of data **7**

3 Numerical integration and parameter estimation **8**

4 Additional model features **9**

4.1 Bachmann . . . 10

4.2 Becker . . . 10

4.3 Beer . . . 10

4.4 Brannmark . . . 11

4.5 Chen . . . 11

4.6 Crauste . . . 11

4.7 Fujita . . . 12

4.8 Hass . . . 12

4.9 Merkle 12
4.10 Sobotta 12
4.11 Swameye 12
4.12 Weber 13
4.13 Zheng 13

5 Waterfall- and scatter plots for the benchmark models 13

6 Example of turnover reaction 34

7 Impact of parameter transformation of the convexity of the objective function 35

8 Number of converged local optimization runs 37

9 Influence of threshold for defining convergence 38

10 Correlation of number of function evaluations and iterations 40

11 Optimization results on simulated data 41

12 Identifiability analysis 42

13 Analysis of sloppiness 43

1 Detailed description of provided files

In this section, an overview of the human-readable output that is available for each benchmark model will be given. This comprises a general overview, and condition-specific data and model files. In the git repository Benchmarking-Initiative/Benchmark-Models, MATLAB scripts are available to automatically read and simulate the models, and for performing a comparison to the supplied model values of our simulation.

1.1 General overview

The Excel files for model information comprise a general information, where the name of the model, its number of data points, parameters and experimental conditions are summarized, cf. Fig. S1a. In addition, the likelihood value of the best fit, if an error model is estimated, and the χ^2 value with fixed uncertainties are provided. Details can be found in Eqs. (1), (2). Moreover, the different model compartments and possible non-trivial model features are summarized. In the second sheet, all model parameters, their values at the best fit, potential logarithmic transformation, bounds and flags indicating whether they are estimated or fixed in the original model are provided (Fig. S1b).

The second to last sheet includes a comprehensive table of the distinct experimental conditions with their individual parameter assignments, number of time and data points and χ^2 value (Fig. S2). The predictor column indicates whether a different predictor than time is used, for example in a dose-response relationship. The parameter assignments are either fixed values or functions of other model parameters. The table is split into assignments that are condition-specific and differ between data files, noted in the first column, and model-wide transformations that are summarized in the lower part.

This information can be used together with the last sheet, which lists all original ordinary differential equations (ODEs) before assignments are applied (Fig. S3). The left- and right-hand sides are stated in distinct columns

	A	B
1	Model:	Bachmann_MSB2011
2		
3	FACTS	
4	The model contains 542 data points, 113 free parameters and 23 experimental conditions	
5	Errors are assumend as additive Gaussian errors	
6		
7	Likelihood value of the model is	-478.4596892
8	Chi2 value of the model is	541.0020223
9		
10	Compartments	size
11	cyt	0.4
12	nuc	0.275
13		
14	Special features of the model	
15	Splines	no
16	Numerical steady-state equilibration	no
17	log-scale of observations	yes
18	Events	0
19	Number of parameter dependent inital conditions	8
20		
21		
22		
23		
24		
25		

General Info | Parameters | Experimental conditions | Raw ODEs

(a) General informations about the model

	A	B	C	D	E	F
1	parameter	value	lower boun	upper boun	analysis at l	estimated
2	log10(CISEqc)	2.636341463	-3	4	1	yes
3	log10(CISEqcOE)	-0.275501067	-3	3	1	yes
4	log10(CISInh)	8.894991931	-3	12	1	yes
5	log10(CISRNADelay)	-0.839300398	-3	3	1	yes
6	log10(CISRNAEqc)	0	-3	3	1	fixed
7	log10(CISRNATurn)	3	-3	3	1	yes
8	log10(CISTurn)	-2.075781466	-3	3	1	yes
9	log10(EpoRActJAK2)	-0.573001594	-3	4	1	yes
10	log10(EpoRCISInh)	6	-3	6	1	yes
11	log10(EpoRCISRemove)	0.734791258	-3	3	1	yes
12	log10(JAK2ActEpo)	5.801509499	-3	9	1	yes
13	log10(JAK2EpoRDeaSHP1)	2.154494177	-3	4	1	yes
14	log10(SHP1ActEpoR)	-3	-3	3	1	yes
15	log10(SHP1Dea)	-2.088189828	-3	3	1	yes
16	log10(SHP1ProOE)	0.451123028	-3	3	1	yes
17	log10(SOCS3Eqc)	2.239652166	-3	3	1	yes
18	log10(SOCS3EqcOE)	-0.168011368	-3	3	1	yes
19	log10(SOCS3Inh)	1.017343315	-3	3	1	yes
20	log10(SOCS3RNADelay)	0.027162044	-3	3	1	yes
21	log10(SOCS3RNAEqc)	0	-3	3	1	fixed
22	log10(SOCS3RNATurn)	-2.080437462	-3	3	1	yes
23	log10(SOCS3Turn)	4	-3	4	1	yes
24	log10(STAT5ActEpoR)	1.59103426	-3	3	1	yes
25	log10(STAT5ActJAK2)	-1.107299849	-3	3	1	yes

General Info | Parameters | Experimental conditions | Raw ODEs

(b) List of parameters of the model, including their values, boundaries and information if they are estimated and on log scale.

Supplementary Figure S1: Screenshots of general model information.

to simplify machine-readability. To obtain condition-specific ODEs that can be compared to the corresponding data, the specific assignments of the desired data set as well as the model-wide assignments have to be applied. Thereby, only one substitution is required, since no parameters should appear within the formulas that need to be replaced themselves. In rare cases, integration time also has to be replaced by the predictor that enters the ODEs. In addition, some parameters might require assignments from the definitions section, which are stated below the ODEs in the sheet *Raw ODEs*. After this substitution, all remaining parameters should appear in the sheet *Parameters*, and the data-specific observation functions and initial values for integration can be found in the condition-specific model file, see next subsection.

	A	B	C	D	E	F	G	H	I	J	K	L	M	N	O	P
1	Conditions															
2																
3	Model file 1		exp condition	nTimePoints	Predictor	nDataPoints	chi2 value	ActD	CISoe	SOCS3oe	epo_level	init_CIS	init_EpoRJ	init_SHP1	init_SOCS3	[illegible]
4		Data file 1	1	14	time	73	95.97198208	0	0	0	1.25e-7	0	0	init_SHP1	0	[illegible]
5		Data file 2	1	11	time	20	20.00063621	0	0	0	1.25e-7	0	0	init_SHP1	0	
6		Data file 3	1	6	time	34	33.99994756	0	0	0	1.25e-7	0	0	init_SHP1	0	
7		Data file 4	1	9	time	45	44.5523808	0	0	0	1.25e-7	0	0	init_SHP1	0	
8		Data file 5	2	9	time	45	39.22995935	1	0	0	1.25e-7	0	0	init_SHP1	0	
9		Data file 6	3	30	time	60	81.14271745	0	0	0	1.25e-6	0	0	init_SHP1	0	
10		Data file 7	4	4	time	20	17.79909302	0	0	0	1.25e-7	0 * CISEqcOE * CISEqc	0	init_SHP1	0	
11		Data file 8	5	4	time	20	18.88065392	0	1	0	1.25e-7	1 * CISEqcOE * CISEqc	1	init_SHP1	0	
12		Data file 9	4	5	time	5	1.959430711	0	0	0	1.25e-7	0 * CISEqcOE * CISEqc	0	init_SHP1	0	
13		Data file 10	5	5	time	5	2.559947178	0	1	0	1.25e-7	1 * CISEqcOE * CISEqc	1	init_SHP1	0	
14		Data file 11	6	4	time	20	21.50675623	0	0	0	1.25e-7	0	0	init_SHP1	0 * SOCS3EqcOE * SOCS3Eqc	
15		Data file 12	7	4	time	20	29.34734405	0	0	1	1.25e-7	0	0	init_SHP1	1 * SOCS3EqcOE * SOCS3Eqc	
16		Data file 13	8	5	time	30	17.02317128	0	0	0	1.25e-7	0	0	init_SHP1	0	
17		Data file 14	9	5	time	30	14.06948229	0	0	0	1.25e-7	0	0	init_SHP1	0	
18		Data file 15	10	3	time	6	3.517770764	0	0	0	2.5e-05	0	0	init_SHP1	0	
19		Data file 16	11	3	time	6	0.956260606	0	0	0	2.5e-06	0	0	init_SHP1	0	
20		Data file 17	12	3	time	6	3.61884276	0	0	0	2.5e-07	0	0	init_SHP1	0	
21		Data file 18	13	3	time	6	2.733979264	0	0	0	2.5e-08	0	0	init_SHP1	0	
22		Data file 19	14	3	time	6	1.880644751	0	0	0	2.5e-09	0	0	init_SHP1	0	
23		Data file 20	15	3	time	6	3.986955398	0	0	0	1.25e-06	0	0	init_SHP1	0	
24		Data file 21	16	3	time	6	7.968874807	0	0	0	1.25e-07	0	0	init_SHP1	0	
25		Data file 22	11	3	time	6	4.88942716	0	0	0	2.5e-06	0	0	init_SHP1	0	
26		Data file 23	12	3	time	6	3.154729878	0	0	0	2.5e-07	0	0	init_SHP1	0	
27		Data file 24	13	3	time	6	3.852462559	0	0	0	2.5e-08	0	0	init_SHP1	0	
28		Data file 25	14	3	time	6	6.59924506	0	0	0	2.5e-09	0	0	init_SHP1	0	
29		Data file 26	16	3	time	3	0.246911177	0	0	0	1.25e-07	0	0	init_SHP1	0	
30		Data file 27	17	3	time	3	10.09332509	0	0	0	1.25e-08	0	0	init_SHP1	0	
31		Data file 28	18	1	time	0	0	0	0	0	1.75e-08	0	0	init_SHP1	0	
32		Data file 29	19	1	time	0	0	0	0	0	1.7675e-07	0	0	init_SHP1	0	
33		Data file 30	11	3	time	3	0.671395245	0	0	0	2.5e-06	0	0	init_SHP1	0	
34		Data file 31	12	3	time	3	0.751156589	0	0	0	2.5e-07	0	0	init_SHP1	0	
35		Data file 32	13	3	time	3	5.817904357	0	0	0	2.5e-08	0	0	init_SHP1	0	
36		Data file 33	14	3	time	3	15.16748791	0	0	0	2.5e-09	0	0	init_SHP1	0	
37		Data file 34	20	1	time	0	0	0	0	0	3.95e-08	0	0	init_SHP1	0	
38		Data file 35	21	1	time	0	0	0	0	0	7.905e-07	0	0	init_SHP1	0	
39		Data file 36	22	1	time	0	0	0	0	0	7.905e-09	0	0	init_SHP1	0	
40		Data file 37	23	1	time	0	0	0	0	0	7.9e-08	0	0	init_SHP1	0	
41		Data file 38	16	3	time	6	2.229044694	0	0	0	1.25e-07	0	0	init_SHP1	0	
42		Data file 39	11	3	time	6	2.661311642	0	0	0	2.5e-06	0	0	init_SHP1	0	
43		Data file 40	12	3	time	6	1.916538932	0	0	0	2.5e-07	0	0	init_SHP1	0	
44		Data file 41	13	3	time	6	16.51044693	0	0	0	2.5e-08	0	0	init_SHP1	0	
45		Data file 42	14	3	time	6	3.733804635	0	0	0	2.5e-09	0	0	init_SHP1	0	
46																
47																
48	General transformations															
49	Parameter	Replacement														
50	CISEqc	CISEqc / CISRNAEqc														
51	CISEqcOE	CISEqcOE * CISEqc														

General Info | Parameters | Experimental conditions | Raw ODEs | +

Supplementary Figure S2: Table of different experimental conditions within a model with corresponding parameter assignments.

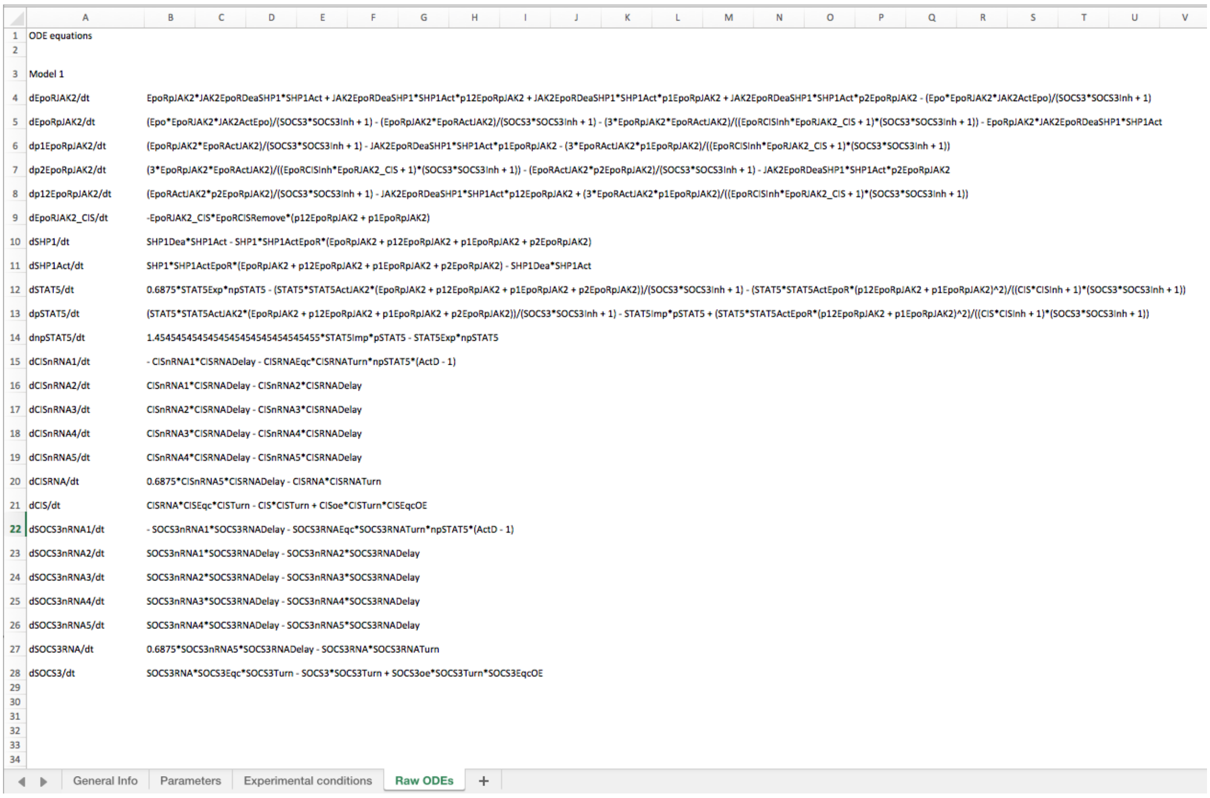

	A	B
1	ODE equations	
2		
3	Model 1	
4	dEpoRJAK2/dt	EpoRpJAK2*JAK2EpoRDeaSHP1*SHP1Act + JAK2EpoRDeaSHP1*SHP1Act*p12EpoRpJAK2 + JAK2EpoRDeaSHP1*SHP1Act*p1EpoRpJAK2 + JAK2EpoRDeaSHP1*SHP1Act*p2EpoRpJAK2 - (Epo*EpoRJAK2*JAK2ActEpo)/(SOCS3*SOCS3Inh + 1)
5	dEpoRpJAK2/dt	(Epo*EpoRJAK2*JAK2ActEpo)/(SOCS3*SOCS3Inh + 1) - (EpoRpJAK2*EpoRActJAK2)/(SOCS3*SOCS3Inh + 1) - (3*EpoRpJAK2*EpoRActJAK2)/((EpoRCISInh*EpoRJAK2_CIS + 1)*(SOCS3*SOCS3Inh + 1)) - EpoRpJAK2*JAK2EpoRDeaSHP1*SHP1Act
6	dp1EpoRpJAK2/dt	(EpoRpJAK2*EpoRActJAK2)/(SOCS3*SOCS3Inh + 1) - JAK2EpoRDeaSHP1*SHP1Act*p1EpoRpJAK2 - (3*EpoRActJAK2*p1EpoRpJAK2)/((EpoRCISInh*EpoRJAK2_CIS + 1)*(SOCS3*SOCS3Inh + 1))
7	dp2EpoRpJAK2/dt	(3*EpoRpJAK2*EpoRActJAK2)/((EpoRCISInh*EpoRJAK2_CIS + 1)*(SOCS3*SOCS3Inh + 1)) - (EpoRActJAK2*p2EpoRpJAK2)/(SOCS3*SOCS3Inh + 1) - JAK2EpoRDeaSHP1*SHP1Act*p2EpoRpJAK2
8	dp12EpoRpJAK2/dt	(EpoRActJAK2*p2EpoRpJAK2)/(SOCS3*SOCS3Inh + 1) - JAK2EpoRDeaSHP1*SHP1Act*p12EpoRpJAK2 + (3*EpoRActJAK2*p1EpoRpJAK2)/((EpoRCISInh*EpoRJAK2_CIS + 1)*(SOCS3*SOCS3Inh + 1))
9	dEpoRJAK2_CIS/dt	-EpoRJAK2_CIS*EpoRCISRemove*(p12EpoRpJAK2 + p1EpoRpJAK2)
10	dSHP1/dt	SHP1Dea*SHP1Act - SHP1*SHP1ActEpoR*(EpoRpJAK2 + p12EpoRpJAK2 + p1EpoRpJAK2 + p2EpoRpJAK2)
11	dSHP1Act/dt	SHP1*SHP1ActEpoR*(EpoRpJAK2 + p12EpoRpJAK2 + p1EpoRpJAK2 + p2EpoRpJAK2) - SHP1Dea*SHP1Act
12	dSTAT5/dt	0.6875*STAT5Exp*npSTAT5 - (STAT5*STAT5ActJAK2*(EpoRpJAK2 + p12EpoRpJAK2 + p1EpoRpJAK2 + p2EpoRpJAK2))/(SOCS3*SOCS3Inh + 1) - (STAT5*STAT5ActEpoR*(p12EpoRpJAK2 + p1EpoRpJAK2)^2)/((CIS*CISInh + 1)*(SOCS3*SOCS3Inh + 1))
13	dpSTAT5/dt	(STAT5*STAT5ActJAK2*(EpoRpJAK2 + p12EpoRpJAK2 + p1EpoRpJAK2 + p2EpoRpJAK2))/(SOCS3*SOCS3Inh + 1) - STAT5Imp*pSTAT5 + (STAT5*STAT5ActEpoR*(p12EpoRpJAK2 + p1EpoRpJAK2)^2)/((CIS*CISInh + 1)*(SOCS3*SOCS3Inh + 1))
14	dnpSTAT5/dt	1.4545454545454545454545454545455*STAT5Imp*pSTAT5 - STAT5Exp*npSTAT5
15	dCISnRNA1/dt	- CISnRNA1*CISRNADelay - CISRNAEqc*CISRNATurn*npSTAT5*(ActD - 1)
16	dCISnRNA2/dt	CISnRNA1*CISRNADelay - CISnRNA2*CISRNADelay
17	dCISnRNA3/dt	CISnRNA2*CISRNADelay - CISnRNA3*CISRNADelay
18	dCISnRNA4/dt	CISnRNA3*CISRNADelay - CISnRNA4*CISRNADelay
19	dCISnRNA5/dt	CISnRNA4*CISRNADelay - CISnRNA5*CISRNADelay
20	dCISRNA/dt	0.6875*CISnRNA5*CISRNADelay - CISRNA*CISRNATurn
21	dCIS/dt	CISRNA*CISEqc*CISTurn - CIS*CISTurn + CISoe*CISTurn*CISEqcOE
22	dSOCS3nRNA1/dt	- SOCS3nRNA1*SOCS3RNADelay - SOCS3RNAEqc*SOCS3RNATurn*npSTAT5*(ActD - 1)
23	dSOCS3nRNA2/dt	SOCS3nRNA1*SOCS3RNADelay - SOCS3nRNA2*SOCS3RNADelay
24	dSOCS3nRNA3/dt	SOCS3nRNA2*SOCS3RNADelay - SOCS3nRNA3*SOCS3RNADelay
25	dSOCS3nRNA4/dt	SOCS3nRNA3*SOCS3RNADelay - SOCS3nRNA4*SOCS3RNADelay
26	dSOCS3nRNA5/dt	SOCS3nRNA4*SOCS3RNADelay - SOCS3nRNA5*SOCS3RNADelay
27	dSOCS3RNA/dt	0.6875*SOCS3nRNA5*SOCS3RNADelay - SOCS3RNA*SOCS3RNATurn
28	dSOCS3/dt	SOCS3RNA*SOCS3Eqc*SOCS3Turn - SOCS3*SOCS3Turn + SOCS3oe*SOCS3Turn*SOCS3EqcOE
29		
30		
31		
32		
33		
34		

Supplementary Figure S3: ODEs of the model before any parameter assignments were applied.

1.2 Condition-specific model files

Modeling problems can contain a large set of experimental conditions with distinct dynamics which are possibly linked to multiple data sets. In order to provide a simple and clear assignment of model equations for each data set, we provide the respective mechanistic model individually for each data file. The files report in the first sheet the corresponding ODEs (after the execution of the experiment-specific parameter assignments listed in the general overview, see Fig. S4). Similar to the general overview, the left- and right-hand sides are printed in different columns. Steps at discrete time points are denoted by heaviside functions that can be readily used within MATLAB.

Moreover, initial values $x(0)$, which are either given by a parameter, a function representing an analytical steady state or a fixed value are listed (Fig. S5b). The first entry specifies the starting point that has to be set for numerical integration algorithms. In addition, the observation functions that map ODE solutions to the data are given, whereby a possible log scale, information about simultaneously estimated uncertainties and the error model are stated. Note that for observations on the log scale, a single absolute error parameter corresponds to a relative error parameter on the original data. Auxiliary definitions, e.g. a sum of all modifications of a protein measured as a total protein concentrations, are listed as well (Fig. S5a).

The information provided for each data set individually can directly be used to simulate the model using standard ODE solvers and to obtain model trajectories that can be compared to the particular data set. Tables providing the experimental data as well as model predictions which are valuable for testing correct implementation are stated in the following.

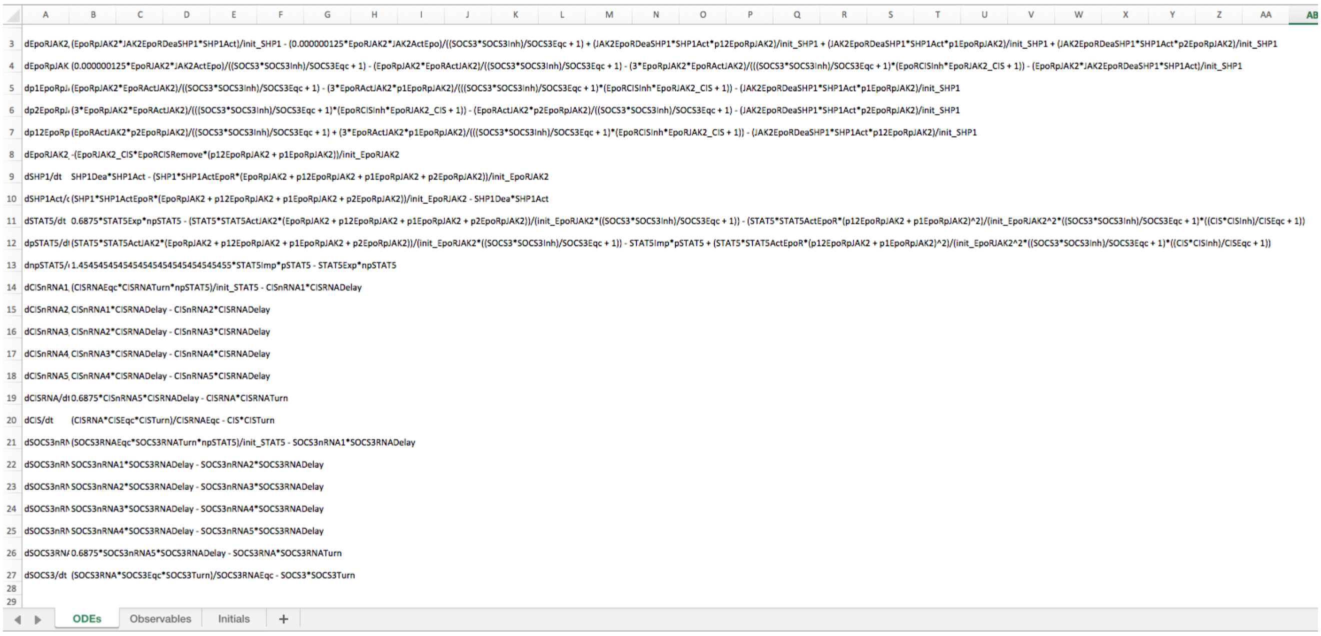

	A	B
3	dEpoRJAK2_	(EpoRpJAK2*JAK2EpoRDeaSHP1*SHP1Act)/init_SHP1 - (0.000000125*EpoRJAK2*JAK2ActEpo)/((SOCS3*SOCS3Inh)/SOCS3Eqc + 1) + (JAK2EpoRDeaSHP1*SHP1Act*p12EpoRpJAK2)/init_SHP1 + (JAK2EpoRDeaSHP1*SHP1Act*p1EpoRpJAK2)/init_SHP1 + (JAK2EpoRDeaSHP1*SHP1Act*p2EpoRpJAK2)/init_SHP1
4	dEpoRpJAK	(0.000000125*EpoRJAK2*JAK2ActEpo)/((SOCS3*SOCS3Inh)/SOCS3Eqc + 1) - (EpoRpJAK2*EpoRActJAK2)/((SOCS3*SOCS3Inh)/SOCS3Eqc + 1) - (3*EpoRpJAK2*EpoRActJAK2)/(((SOCS3*SOCS3Inh)/SOCS3Eqc + 1)*(EpoRCISInh*EpoRJAK2_CIS + 1)) - (EpoRpJAK2*JAK2EpoRDeaSHP1*SHP1Act)/init_SHP1
5	dp1EpoRpJ	(EpoRpJAK2*EpoRActJAK2)/((SOCS3*SOCS3Inh)/SOCS3Eqc + 1) - (3*EpoRActJAK2*p1EpoRpJAK2)/(((SOCS3*SOCS3Inh)/SOCS3Eqc + 1)*(EpoRCISInh*EpoRJAK2_CIS + 1)) - (JAK2EpoRDeaSHP1*SHP1Act*p1EpoRpJAK2)/init_SHP1
6	dp2EpoRpJ	(3*EpoRpJAK2*EpoRActJAK2)/(((SOCS3*SOCS3Inh)/SOCS3Eqc + 1)*(EpoRCISInh*EpoRJAK2_CIS + 1)) - (EpoRActJAK2*p2EpoRpJAK2)/((SOCS3*SOCS3Inh)/SOCS3Eqc + 1) - (JAK2EpoRDeaSHP1*SHP1Act*p2EpoRpJAK2)/init_SHP1
7	dp12EpoRp	(EpoRActJAK2*p2EpoRpJAK2)/((SOCS3*SOCS3Inh)/SOCS3Eqc + 1) + (3*EpoRActJAK2*p1EpoRpJAK2)/(((SOCS3*SOCS3Inh)/SOCS3Eqc + 1)*(EpoRCISInh*EpoRJAK2_CIS + 1)) - (JAK2EpoRDeaSHP1*SHP1Act*p12EpoRpJAK2)/init_SHP1
8	dEpoRJAK2_	-(EpoRJAK2_CIS*EpoRCISRemove*(p12EpoRpJAK2 + p1EpoRpJAK2))/init_EpoRJAK2
9	dSHP1/dt	SHP1Dea*SHP1Act - (SHP1*SHP1ActEpoR*(EpoRpJAK2 + p12EpoRpJAK2 + p1EpoRpJAK2 + p2EpoRpJAK2))/init_EpoRJAK2
10	dSHP1Act/d	(SHP1*SHP1ActEpoR*(EpoRpJAK2 + p12EpoRpJAK2 + p1EpoRpJAK2 + p2EpoRpJAK2))/init_EpoRJAK2 - SHP1Dea*SHP1Act
11	dSTAT5/dt	0.6875*STAT5Exp*npSTAT5 - (STAT5*STAT5ActJAK2*(EpoRpJAK2 + p12EpoRpJAK2 + p1EpoRpJAK2 + p2EpoRpJAK2))/(init_EpoRJAK2*((SOCS3*SOCS3Inh)/SOCS3Eqc + 1)) - (STAT5*STAT5ActEpoR*(p12EpoRpJAK2 + p1EpoRpJAK2)^2)/(init_EpoRJAK2^2*((SOCS3*SOCS3Inh)/SOCS3Eqc + 1)*((CIS*CISInh)/CISEqc + 1))
12	dpSTAT5/dt	(STAT5*STAT5ActJAK2*(EpoRpJAK2 + p12EpoRpJAK2 + p1EpoRpJAK2 + p2EpoRpJAK2))/(init_EpoRJAK2*((SOCS3*SOCS3Inh)/SOCS3Eqc + 1)) - STAT5Imp*pSTAT5 + (STAT5*STAT5ActEpoR*(p12EpoRpJAK2 + p1EpoRpJAK2)^2)/(init_EpoRJAK2^2*((SOCS3*SOCS3Inh)/SOCS3Eqc + 1)*((CIS*CISInh)/CISEqc + 1))
13	dnpSTAT5/	1.45454545454545454545454545455*STAT5Imp*pSTAT5 - STAT5Exp*npSTAT5
14	dCISnRNA1	(CISRNAEqc*CISRNATurn*npSTAT5)/init_STAT5 - CISnRNA1*CISRNADelay
15	dCISnRNA2	CISnRNA1*CISRNADelay - CISnRNA2*CISRNADelay
16	dCISnRNA3	CISnRNA2*CISRNADelay - CISnRNA3*CISRNADelay
17	dCISnRNA4	CISnRNA3*CISRNADelay - CISnRNA4*CISRNADelay
18	dCISnRNA5	CISnRNA4*CISRNADelay - CISnRNA5*CISRNADelay
19	dCISRNA/dt	0.6875*CISnRNA5*CISRNADelay - CISRNA*CISRNATurn
20	dCIS/dt	(CISRNA*CISEqc*CISTurn)/CISRNAEqc - CIS*CISTurn
21	dSOCS3nRN	(SOCS3RNAEqc*SOCS3RNATurn*npSTAT5)/init_STAT5 - SOCS3nRNA1*SOCS3RNADelay
22	dSOCS3nRN	SOCS3nRNA1*SOCS3RNADelay - SOCS3nRNA2*SOCS3RNADelay
23	dSOCS3nRN	SOCS3nRNA2*SOCS3RNADelay - SOCS3nRNA3*SOCS3RNADelay
24	dSOCS3nRN	SOCS3nRNA3*SOCS3RNADelay - SOCS3nRNA4*SOCS3RNADelay
25	dSOCS3nRN	SOCS3nRNA4*SOCS3RNADelay - SOCS3nRNA5*SOCS3RNADelay
26	dSOCS3RNA	0.6875*SOCS3nRNA5*SOCS3RNADelay - SOCS3RNA*SOCS3RNATurn
27	dSOCS3/dt	(SOCS3RNA*SOCS3Eqc*SOCS3Turn)/SOCS3RNAEqc - SOCS3*SOCS3Turn

ODEs | Observables | Initials

Supplementary Figure S4: ODEs of the model after condition-specific parameter assignments are applied.

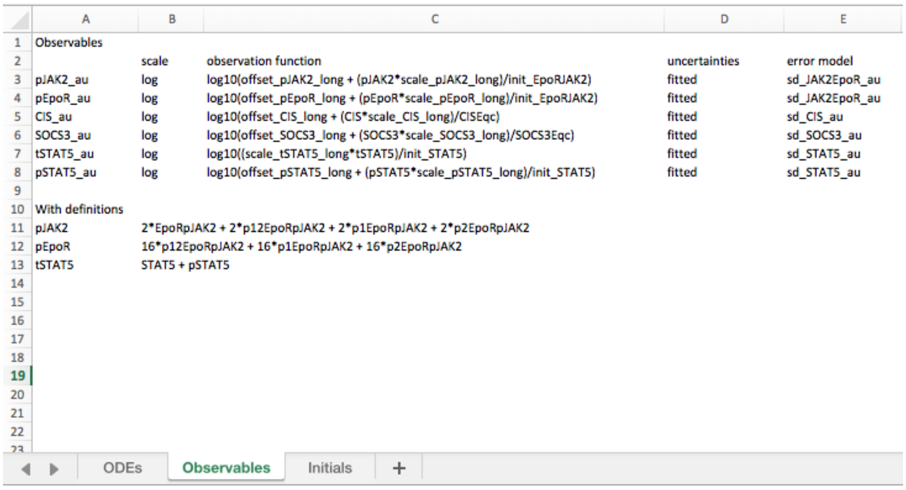

	A	B	C	D	E
1	Observables				
2		scale	observation function	uncertainties	error model
3	pJAK2_au	log	log10(offset_pJAK2_long + (pJAK2*scale_pJAK2_long)/init_EpoRJAK2)	fitted	sd_JAK2EpoR_au
4	pEpoR_au	log	log10(offset_pEpoR_long + (pEpoR*scale_pEpoR_long)/init_EpoRJAK2)	fitted	sd_JAK2EpoR_au
5	CIS_au	log	log10(offset_CIS_long + (CIS*scale_CIS_long)/CISEqc)	fitted	sd_CIS_au
6	SOCS3_au	log	log10(offset_SOCS3_long + (SOCS3*scale_SOCS3_long)/SOCS3Eqc)	fitted	sd_SOCS3_au
7	tSTAT5_au	log	log10((scale_tSTAT5_long*tSTAT5)/init_STAT5)	fitted	sd_STAT5_au
8	pSTAT5_au	log	log10(offset_pSTAT5_long + (pSTAT5*scale_pSTAT5_long)/init_STAT5)	fitted	sd_STAT5_au
9					
10	With definitions				
11	pJAK2	2*EpoRpJAK2 + 2*p12EpoRpJAK2 + 2*p1EpoRpJAK2 + 2*p2EpoRpJAK2			
12	pEpoR	16*p12EpoRpJAK2 + 16*p1EpoRpJAK2 + 16*p2EpoRpJAK2			
13	tSTAT5	STAT5 + pSTAT5			

ODEs | Observables | Initials

(a) "Observables" sheet contains labels indicating analysis at the log scale as well as equations defining observation functions and error models. Possible definitions of auxiliary variables are denoted as well.

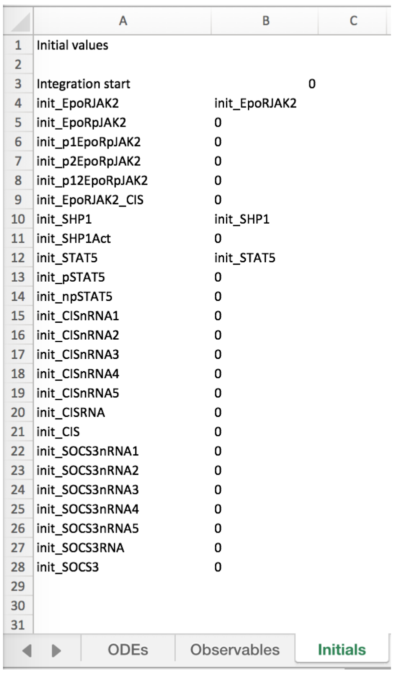

	A	B
1	Initial values	
2		
3	Integration start	0
4	init_EpoRJAK2	init_EpoRJAK2
5	init_EpoRpJAK2	0
6	init_p1EpoRpJAK2	0
7	init_p2EpoRpJAK2	0
8	init_p12EpoRpJAK2	0
9	init_EpoRJAK2_CIS	0
10	init_SHP1	init_SHP1
11	init_SHP1Act	0
12	init_STAT5	init_STAT5
13	init_pSTAT5	0
14	init_npSTAT5	0
15	init_CISnRNA1	0
16	init_CISnRNA2	0
17	init_CISnRNA3	0
18	init_CISnRNA4	0
19	init_CISnRNA5	0
20	init_CISRNA	0
21	init_CIS	0
22	init_SOCS3nRNA1	0
23	init_SOCS3nRNA2	0
24	init_SOCS3nRNA3	0
25	init_SOCS3nRNA4	0
26	init_SOCS3nRNA5	0
27	init_SOCS3RNA	0
28	init_SOCS3	0

ODEs | Observables | Initials

(b) Initial values of the ODEs for all model states.

Supplementary Figure S5: Screenshots of a model specific human readable definition file, provided as Excel file.

1.3 Condition-specific data files

The data for individual experiments are provided as separate Excel files. File names and enumeration correspond to the respective model files and to the condition-specific parameter transformation table provided as

time	pJAK2_au	pJAK2_au_std	pJAK2_au_ExpError	pEpoR_au	pEpoR_au_std	pEpoR_au_ExpError
0	-1.955997626	0.212721623		-2.35532523	0.212721623	
5	0	0.212721623		-0.154400562	0.212721623	
10	-0.127344806	0.212721623		0	0.212721623	
20	-0.127344806	0.212721623		-0.217191392	0.212721623	
40	-0.581607138	0.212721623		-0.719316116	0.212721623	
60	-1.365472629	0.212721623		-0.996595215	0.212721623	
80	-0.787282786	0.212721623		-0.954877441	0.212721623	
100	-1.033352739	0.212721623		-1.155856215	0.212721623	
120	-1.597287476	0.212721623		-0.548184611	0.212721623	
140	-0.87244778	0.212721623		-0.872772917	0.212721623	
160	-0.866920046	0.212721623		-0.620594296	0.212721623	
180	-1.250016128	0.212721623		-1.295146764	0.212721623	
220	-1.227362269	0.212721623		-1.397735201	0.212721623	
240	-0.990906111	0.212721623		-1.068890435	0.212721623	

Exp Data | Simulation

(a) Example table containing experimental measurements including standard deviations representing measurement uncertainties. These standard deviations are either available as part of the experimental outcomes, or are otherwise taken from the fitted error model.

time	pJAK2_au	pEpoR_au	CIS_au
0	-2.023496231	-2.357396691	-1.577410346
5	-0.293237979	-0.019599576	-1.577410175
10	-0.337264472	-0.057256938	-1.577272635
20	-0.619559564	-0.434667489	-1.546962121
40	-0.822816959	-0.739729261	-0.954930319
60	-0.91367231	-0.885855839	-0.432669511
80	-0.955408914	-0.955191736	-0.179713319
100	-0.973073413	-0.984997807	-0.064449901
120	-0.978861907	-0.994873429	
140	-0.979017431	-0.99521131	-0.007494345
160	-0.976809024	-0.991533008	-0.015473954
180	-0.973926068	-0.98670382	-0.032782871
220	-0.968939352	-0.978341171	-0.076147542
240	-0.967261342	-0.975526663	-0.097172405

Exp Data | Simulation

(b) Example sheet with the corresponding model response, i.e., the simulated values for each data point.

Supplementary Figure S6: Screenshots of an experimental data file provided as Excel file.

part of the general information (Fig. S2).

Therein, the measurement time points are listed in the first column, followed by blocks of three columns for each measured observable. Each block provides measured values as first column and two columns for the standard deviations of measurement errors (Fig. S6a). If standard deviations are known from the experiments, they are provided in the third column. If such standard deviations are not available, we provide an estimated standard deviation from the error model in the second column of each block. The information from a fitted error model is only provided in case of absence of an experimental standard deviation. This strategy allows usage of the benchmark problems for approaches requiring measurement uncertainties for all data points.

The likelihood or χ^2 values provided in the general information use the same logic. Moreover, a sheet with the model output at each measurement point is provided, obtained with the provided parameter values (Fig. S6b). If the corresponding observation is on the log-scale (Fig. S5a), the measurements and simulations are provided on the log-scale as well.

The simulated model response can be used to compare and test different algorithms and software implementations. In our case, *CVODES* from the *SUite of Nonlinear and DIfferential/ALgebraic equation Solvers (SUNDIALS)* (Hindmarsh et al., 2005) has been utilized for numerical integration. See Supplementary Section 3 for detailed information about numerical integration and optimization.

2 Usage of models and optional simulation of data

The provided benchmark models are available in the git repository Benchmarking-Initiative/Benchmark-Models together with directly usable MATLAB scripts to simulate the models, and simulated reference time courses which can be used for testing proper implementation in other software environments. Also, extensive information about usage of the models in either Data2Dynamics or with the independent MATLAB scripts

is given in the GitHub Wiki pages.

Besides the condition-specific data files with experimentally measured data, an additional folder with similar files containing data simulated with the parameters of the best fit are provided. For simulation, the estimated standard deviation from the underlying error model, or the measured uncertainty if provided, is specified. The mentioned MATLAB scripts and Data2Dynamics include functions to automatically generate data that mirrors the experimental data concerning time points and number of measurements, but with the given parameters as ground truth. Moreover, an arbitrary noise level can be specified in terms of the estimated noise of the original model.

3 Numerical integration and parameter estimation

No analytic solutions are available for the ODEs of the provided benchmark models. Thus, numerical integration was performed via the *CVODES* integrator of the *SUite of Nonlinear and DIfferential/ALgebraic equation Solvers (SUNDIALS)* suite (Hindmarsh et al., 2005) which is tailored to solving stiff and non-stiff systems and computation of sensitivities. Absolute and relative tolerances were set for all models by default to 10^{-6}, except for the models of Beer, Boehm, Crauste and Sobotta with a value of 10^{-8}. Minimization of the negative log-likelihood was performed by *lsqnonlin* or *fmincon* (Coleman and Li, 1996) which are both available in MATLAB's optimization toolbox. We used MATLAB release R2017a. Thereby, mainly the default configuration settings were chosen. For fmincon, the algorithm-option was chosen as *trust-region-reflective* or *interior-point*, respectively. Additional changes to the default settings comprise:

- In fmincon, user-defined gradient and Hessian were defined to perform Gauss-Newton optimization, equivalent to optimization by the user-defined Jacobian in lsqnonlin.

- The termination tolerance on first-order optimality and function value was set to 0 in order to terminate optimization only due to small parameter changes.

- Termination tolerance for the parameter changes was set to 10^{-6}.

- As subproblem-algorithm, *cg* (conjugate gradient) was chosen for fmincon and $factorization$ for lsqnonlin.

- The maximum number of iterations was set to 10000.

The derivative information required for deterministic optimization was computed via forward sensitivities, i.e., the ODE system was augmented by the appropriate sensitivity equations (Leis and Kramer, 1988) providing first order derivatives of the dynamics with respect to parameters and initial conditions.

Bessel's correction is a general procedure to reduce the bias of the estimated error parameters, if a noise model is estimated simultaneously. However, it is rather rarely applied in Systems Biology and it provokes the undesired property that the result depends on the number of estimated parameters. Thus, for fitting the benchmark models Bessel's correction was omitted to enable the calculation of χ^2 terms within the objective function. Two times the negative log-likelihood

$$-2\log(\mathcal{L}) = \sum_{i=1}^{n_{data}} 2\log(\sqrt{2\pi}) + \log(\sigma_i) + \left(\frac{y_i - g(x(t_i,\theta),\theta)}{\sigma_i}\right)^2 . \quad (1)$$

was used as objective function for parameter estimation and the result after optimization is provided as part of the *general information*. In addition, the best fit for a fixed error model is provided with its χ^2 value,

$$\chi^2 = \sum_{i=1}^{n_{data}} \left(\frac{y_i - g(x(t_i, \theta), \theta)}{\sigma_i} \right)^2 . \tag{2}$$

Minimizing (1) and (2) is equivalent in the case of known experimental errors, which was denoted as Ex in Table 1 of the main manuscript. The estimated and, if known, experimental uncertainties of the measurements are stored in the data Excel files. The files comprise 3 columns per observation, with (1) the experimental data point, (2) the error output from the estimated error model, and (3) the experimental uncertainties if they exist. This enables fitting the models in case an algorithm cannot handle simultaneous error estimation and requires data uncertainties for each data point.

The SBML files contain information about the observation function, and already comprise a log-transformation in case a log-transformation of the data is utilized in the respective benchmark model. Thus, mapping of data to the models provided in SBML format can easily be accomplished.

4 Additional model features

At this point, we provide further implementation details for some benchmark models. For additional and updated descriptions we refer to the GitHub repository and wiki at https://www.benchmarking.uni-freiburg.de/ and https://github.com/Benchmarking-Initiative/Benchmark-Models. For all models, we provide best fit parameters resulting from minimization of (1) which sometimes does not coincide with published parameters.

Several of the described benchmark models utilize input functions, such as step functions or splines. In the case of an external input, events are typically utilized that reset the integrator, and are specified in Table 1 of the main manuscript. A detailed description of event handling can be found in (Fröhlich et al., 2017). Within SBML, splines are provided as cubic functions with fixed parameters in the intervals between knots. In SBML, these parameters are re-set via events on each anchor point, whereas step functions are implemented as `piecewise` functions.

In the human-readable output format, a step function switching from $level1$ to $level2$ at $t = switch\_time$ is described via

$$level1 + (level2 - level1) \cdot heaviside(switch\_time).$$

A cubic interpolation spline with five knot points at $t = 0.0, 5.0, 10.0, 20.0, 60.0$ is denoted by

$$spline\_pos5(t, 0.0, sp1, 5.0, sp2, 10.0, sp3, 20.0, sp4, 60.0, sp5, 0, 0.0) \ ,$$

and the spline parameters $sp1, \ldots, sp5$ are estimated simultaneously with the other model parameters given the experimental data. We provide *spline_pos5* as C and MATLAB/mex implementations to guarantee that calculation of the splines is reproducible (e.g. that exactly the same method and parametrization are used).

4.1 Bachmann

In Bachmann et al. (2011), several reparameterizations were applied compared to the standard rate-equation approach:

• Some rate constants were defined relative to an initial concentration parameter, e.g. $JAK2EpoRDeaSHP1$ relative to $init\_SHP1$ and $EpoRCISRemove$, $SHP1ActEpoR$, $STAT5ActJAK2$ relative to $init\_EpoRJAK2$. Similarly, $CISRNAEqc$ and $SOCS3RNAEqc$ were analyzed relative to $init\_STAT5$.

• $STAT5ActEpoR$ has concentration unit $[1/conc^2]$ before reparametrization and was analyzed relative to $(init\_EpoRJAK2)^2$ to obtain a parameter which is independent of concentration units.

• Overexpression parameters $CISEqcOE$ and $SOCS3EqcOE$ were analyzed as dimensionless fold-factors relative to the respective wild-type parameters.

• In addition, the following substitutions were performed as reparametrization:

$$\begin{aligned} CISInh &\rightarrow CISInh/CISEqc \\ SOCS3Inh &\rightarrow SOCS3Inh/SOCS3Eqc \\ CISEqc &\rightarrow CISEqc/CISRNAEqc \\ SOCS3Eqc &\rightarrow SOCS3Eqc/SOCS3RNAEqc \end{aligned}$$

Model equations before and after substitutions are available in the human readable model definition files.

4.2 Becker

The model published by Becker et al. constitutes of two model components. The first component is an ODE model describing Epo receptor signaling. The second model component is a function which originates from the so-called *Scatchard plot* analysis. This function describes the relationship between bound and free ligand concentrations on the log-log scale. The slope provides an estimate for the number of receptors. In the Becker model, this slope parameter was simultaneously estimated with the remaining model parameters.

Compared to a standard rate-equation approach, two reparametrizations were in applied in the Becker model:

1. The initial state for the Epo receptor concentration was modeled relative to the Epo ligand amount. For this purpose, the initial condition $init\_EpoR$ for Epo receptors is substituted by the product $init\_Epo \cdot init\_EpoR\_rel$.

2. The binding constant of Epo to receptors termed k_{on} was also parameterized relative to the initial Epo ligand amount. For this purpose k_{on} from the standard rate-equation had been substituted with $k_{on}/init\_Epo$.

4.3 Beer

The model published by Beer et al. is characterized by a large number of data points. These data points are the cells' absorbance evaluated every five minutes in the time range $[0, 5, \ldots, 3565]$ min. and occurs as 38

rather smooth and almost continuous time course, each with 714 data points.

Assuming independent measurement noise for each of these data points seems questionable from our point of view. Nevertheless this benchmark model constitutes a valuable test case for this kind of application data.

Within the chosen integrator and optimizer tolerances, the best optimum could not be found repeatedly for the model of Beer et al. which indicates that optimization did not reliably converge to a global optimum without manual fine-tuning (Fig. S9).

4.4 Brannmark

The dynamic equations of the model published by Brannmark et al. is available in the Biomodels Database (Le Novere et al., 2006) where the model is termed BIOMD0000000343 - Brannmark2010 - Insulin Signalling Mifamodel. The data had been published as Supplementary MATLAB workspaces available as Supplementary file of the original publication (Brännmark et al., 2010). The raw data had been generated in triplicates and means and standard errors of the means (SEMs) were analyzed in Brännmark et al. (2010). However, since the raw data was scaled to the range 0-100, several SEMs are zero which yields undefined terms in the log-likelihood (1). In our benchmark model we therefore omitted the published experimental errors and used an individual error parameter for each observable.

4.5 Chen

For the model of Chen et al., we used the SBML-format version from the Biomodels database (Le Novere et al., 2006) termed as BIOMD0000000255 - Chen2009 - ErbB Signaling. This model implements step functions as a sine function to smoothly connect the two levels specified in input functions.

The Chen model has 500 states and 191 parameters which both exceeds the number of data points (N=105). The total number of parameters was reduced with respect to the original publication, since the additional parameters defined in Chen et al. do not take part in the model dynamics. Within the chosen integrator and optimizer tolerances, optimization did not converge. The step in Fig. S13 results from initial parameter values with flat model trajectories due to small scaling factors and does not correspond to an optimum after successful fitting.

A successful optimization requires adjoint sensitivities that are not implemented in Data2Dynamics, see (Villaverde et al., 2018).

4.6 Crauste

Within the chosen integrator and optimizer tolerances, the best optimum could not be found repeatedly for the model of Crauste et al. which indicates that optimization did not reliably converge to a global optimum without manual fine-tuning (Fig. S14). Yet, with adapted optimization settings, the best fit value was found repeatedly.

4.7 Fujita

Within the chosen integrator and optimizer tolerances, the best optimum could not be found repeatedly for the model of Fujita et al. which indicates that optimization did not reliably converge to a global optimum without manual fine-tuning (Fig. S16).

4.8 Hass

The model of Hass et al. was implemented with initialization at negative time points at $t = -30$ min to account for prestimulation settings. If the model should be utilized for strictly positive times, the equations and the measurement times have to be reformulated accordingly.

4.9 Merkle

In the original publication, there are additional model versions and stages which were applied in combination with L1-penalization to select cell-type specific parameters. In this benchmark collection, we used the comprehensive model for CFU-E and H838 cells after identification of cell-type specific parameters which was termed as "parsimonious model" with "final parameter estimates with a non-regularized optimization". Within the Data2Dynamics examples, this model version is termed "Merkle_JAK2STAT5_PCB2016, final model". The model constitutes of two submodels representing both cell types. A number of experimental conditions were added for model analysis and validation, which do not contain data but can be used to simulate the model.

4.10 Sobotta

The model of Sobotta et al. requires integration to be initialized at negative time points at $t = -10$ min. If the model should be utilized for strictly positive times, the equations and the measurement times have to be reformulated accordingly.

4.11 Swameye

The model published by Swameye et al. is available in several public versions. In the original publication (Swameye et al., 2003), a delay-differential equation was applied and the input was defined by linear interpolation between measurements. In (Raue et al., 2009), the delay was replaced by a linear chain and a cubic interpolation spline was applied to estimate the input. In (Schelker et al., 2012) the model was utilized to introduce comprehensive parameter estimation of ODE, observation- and input parameters. Following this progress, the spline is represented by a cubic spline with with five knots at t=0, 5, 10, 20, and 60 min. The parametrization by control points $sp_1, sp_2, \ldots, sp_5$ as used in (Schelker et al., 2012) is utilized and the spline is evaluated at the log-concentration scale. Within this parametrization, the control points represent the estimated function values of the spline at the knot times. The spline parameters are comprehensively estimated with the remaining model parameters. The delay is represented with a five step linear chain which are linked by a single parameter.

Please note that there are several different implementations for cubic splines available. For benchmarking purposes, it is important to qualitatively stick to setup described here but any implementation for cubic

splines might be applied. However, for perfectly reproducing the results of this paper or for comparisons of the obtained value of the objective function after fitting, it is essential to use exactly the same implementation. For this case, we provide appropriate C and MATLAB spline implementation files.

4.12 Weber

The model of Weber et al. was parameterized using relative data for several biochemical species. The relative data provide the ratios of the abundance of biochemical species under different experimental settings, e.g. the ratio of measured intensities at two time points. In the original publication, the corresponding ratios were computed from the simulation results and compared to the measured ratios. As many toolboxes do not support this, we implemented these relative data by introducing scaling constants.

4.13 Zheng

We used the aggregated data of Zheng et al. of the histone modifications, summing over the intermediate states for the labeling of the methyl group. The model directly models the overall methylation kinetics and includes an ODE for each of the 15 considered methylation states.

5 Waterfall- and scatter plots for the benchmark models

In order to assess the performance of numerical optimization utilized to fit the models and estimate the parameters, multi-start optimization has been applied. For each benchmark problem, 1000 random initial guesses were drawn, uniformly distributed within the specified bounds. For each initial guess, optimization was performed and the resulting optimized objective function values were sorted and plotted. Thereby, objective values were shifted in order to obtain an objective function value of 1 for the best fit. The resulting figures are shown in the following.

Following this procedure, reliable optimization is characterized by repeatedly finding the same optima, i.e., the same levels of the objective function which results in "steps" in the resulting plots. Moreover, existence of local optima is indicated by steps at different levels. Because the shape is reminiscent of a waterfall, this kind of depiction has been termed *waterfall plot*.

As a second kind of depiction, we plot the resulting value for the objective function against computation time in order to highlight whether there is a relationship between performance benefits and computational effort.

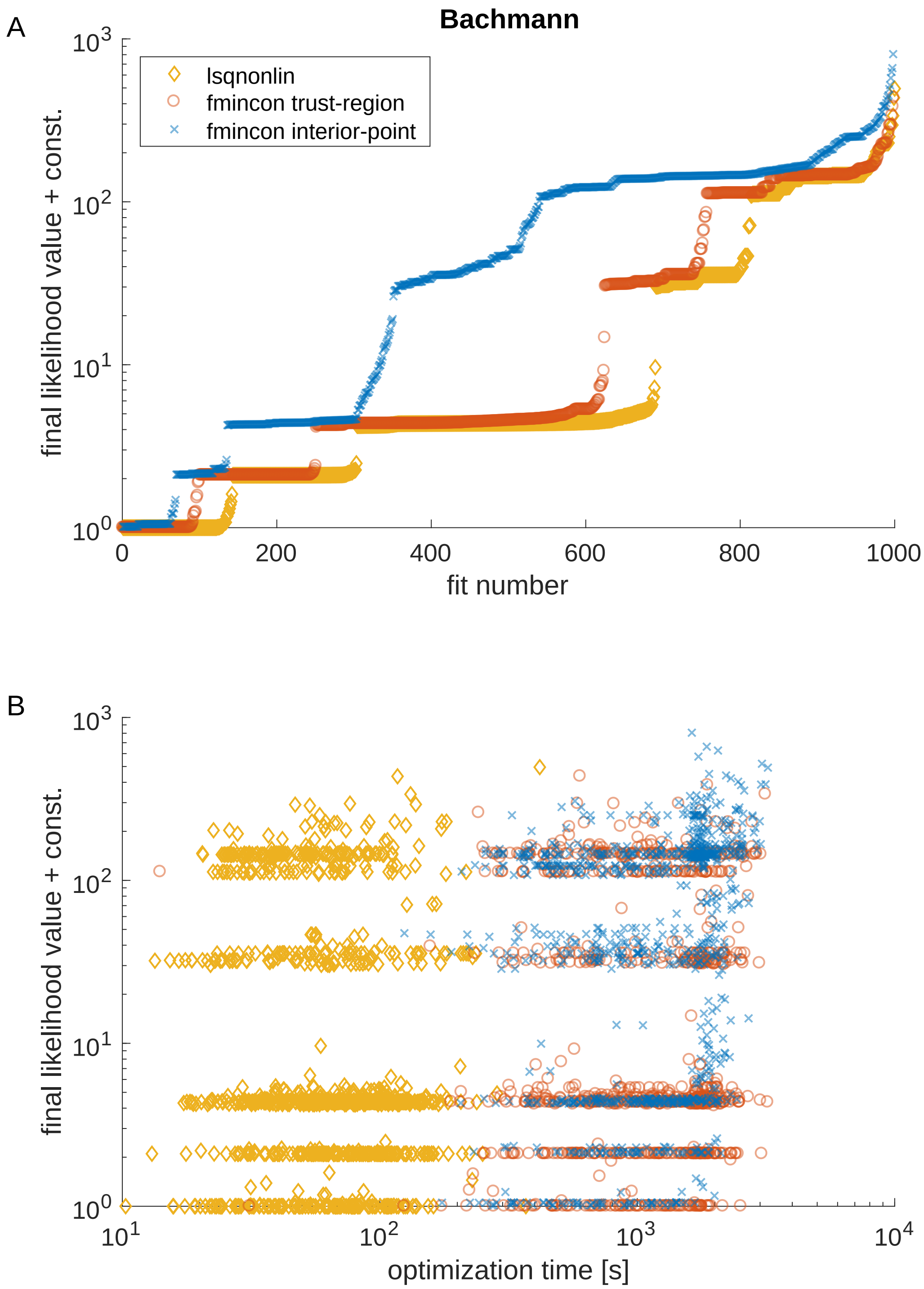

Supplementary Figure S7: (A) Waterfall plot for the Bachmann benchmark model (Bachmann et al., 2011). Sorted likelihood values, with best fit value shifted to 1, are shown on the y-axis for 1000 optimization runs of different optimization algorithms, starting from equal random initial parameter guesses. (B) Scatter plot of the final likelihood values vs. required optimization time.

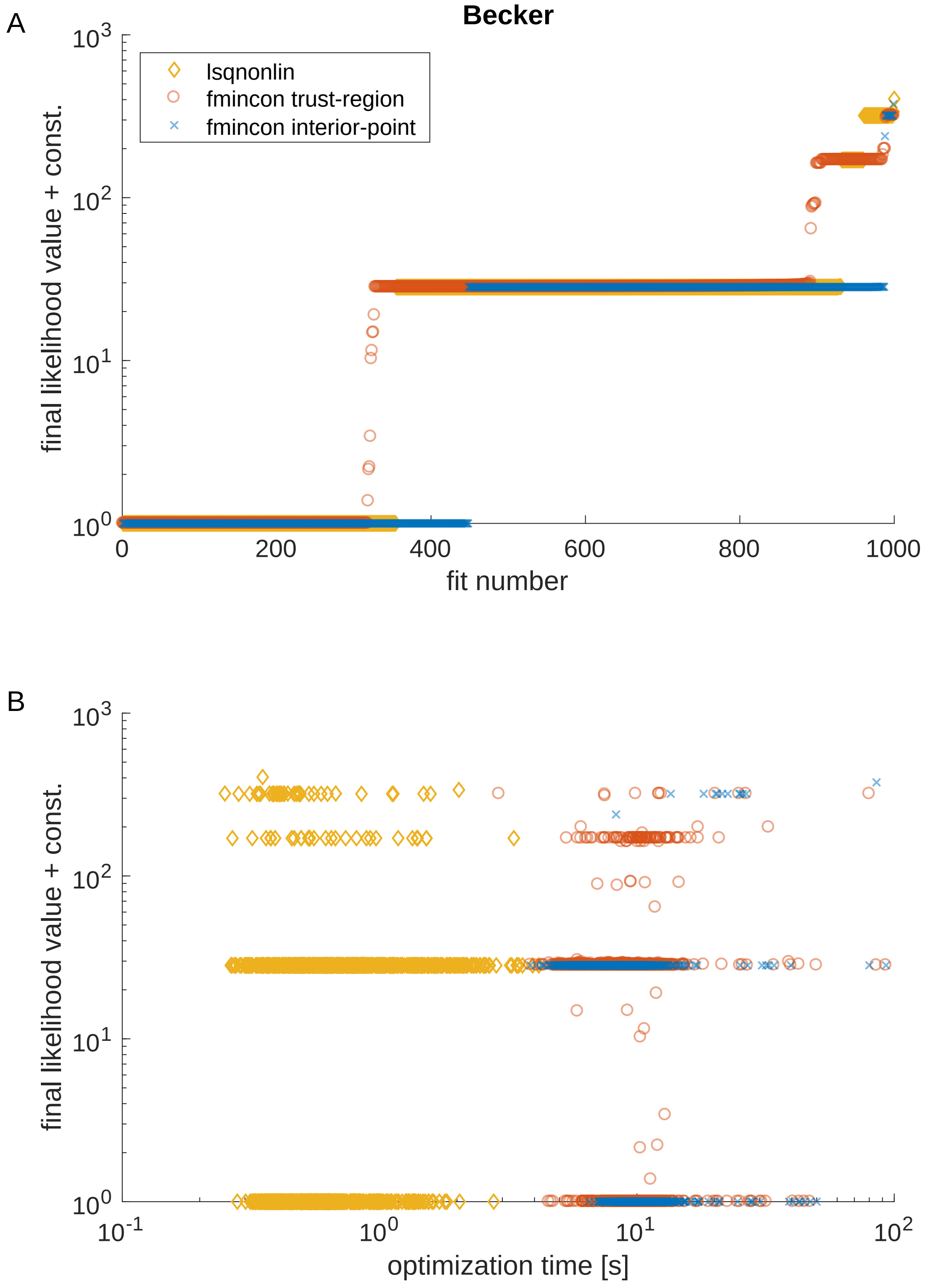

Supplementary Figure S8: (A) Waterfall plot for the Becker benchmark model (Becker et al., 2010). Sorted likelihood values, with best fit value shifted to 1, are shown on the y-axis for 1000 optimization runs of different optimization algorithms, starting from equal random initial parameter guesses. (B) Scatter plot of the final likelihood values vs. required optimization time.

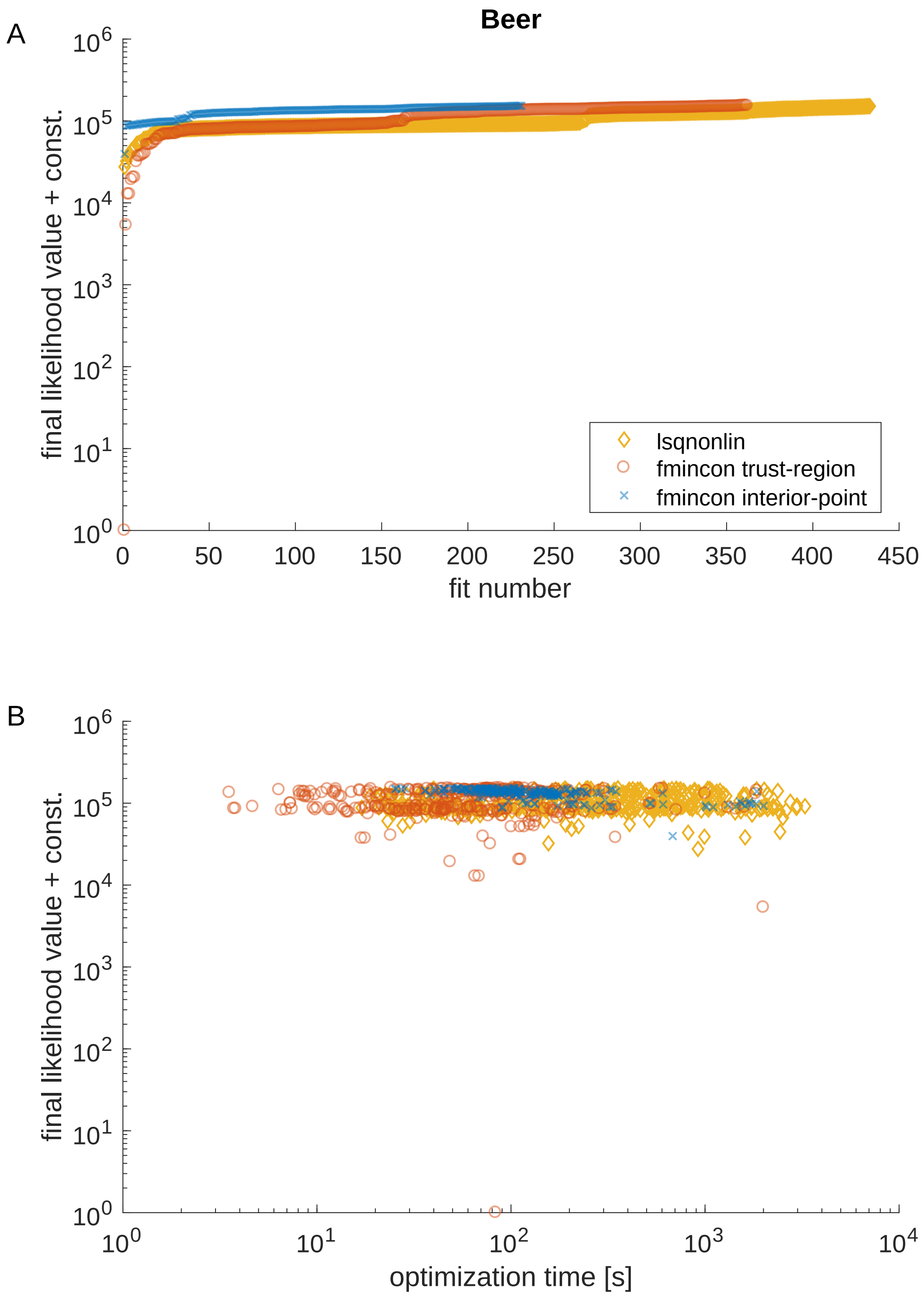

Supplementary Figure S9: (A) Waterfall plot for the Beer benchmark model (Beer et al., 2014). Sorted likelihood values, with best fit value shifted to 1, are shown on the y-axis for 1000 optimization runs of different optimization algorithms, starting from equal random initial parameter guesses. (B) Scatter plot of the final likelihood values vs. required optimization time.

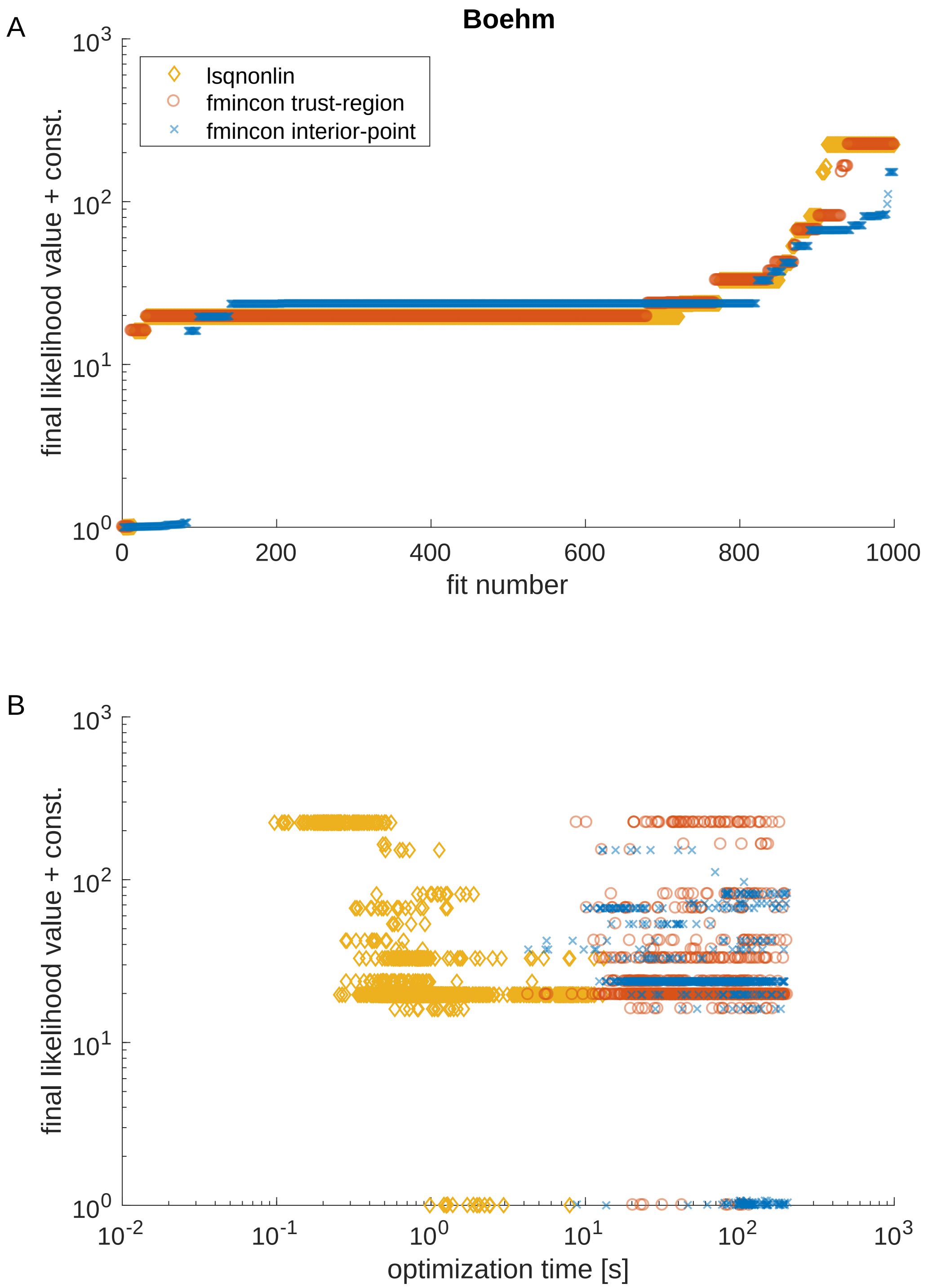

Supplementary Figure S10: (A) Waterfall plot for the Boehm benchmark model (Boehm et al., 2014). Sorted likelihood values, with best fit value shifted to 1, are shown on the y-axis for 1000 optimization runs of different optimization algorithms, starting from equal random initial parameter guesses. (B) Scatter plot of the final likelihood values vs. required optimization time.

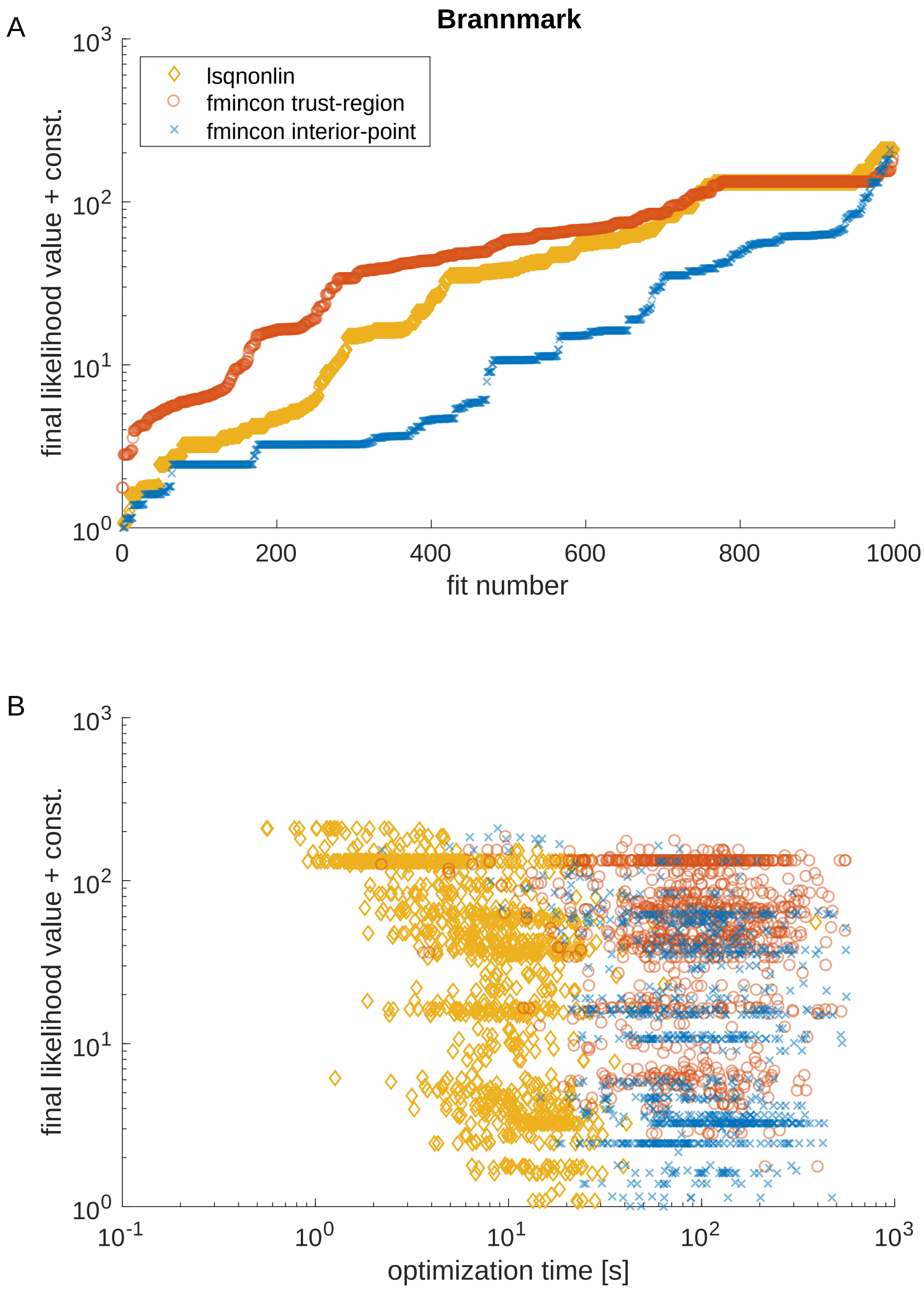

Supplementary Figure S11: (A) Waterfall plot for the Brannmark benchmark model (Brännmark et al., 2010). Sorted likelihood values, with best fit value shifted to 1, are shown on the y-axis for 1000 optimization runs of different optimization algorithms, starting from equal random initial parameter guesses. (B) Scatter plot of the final likelihood values vs. required optimization time.

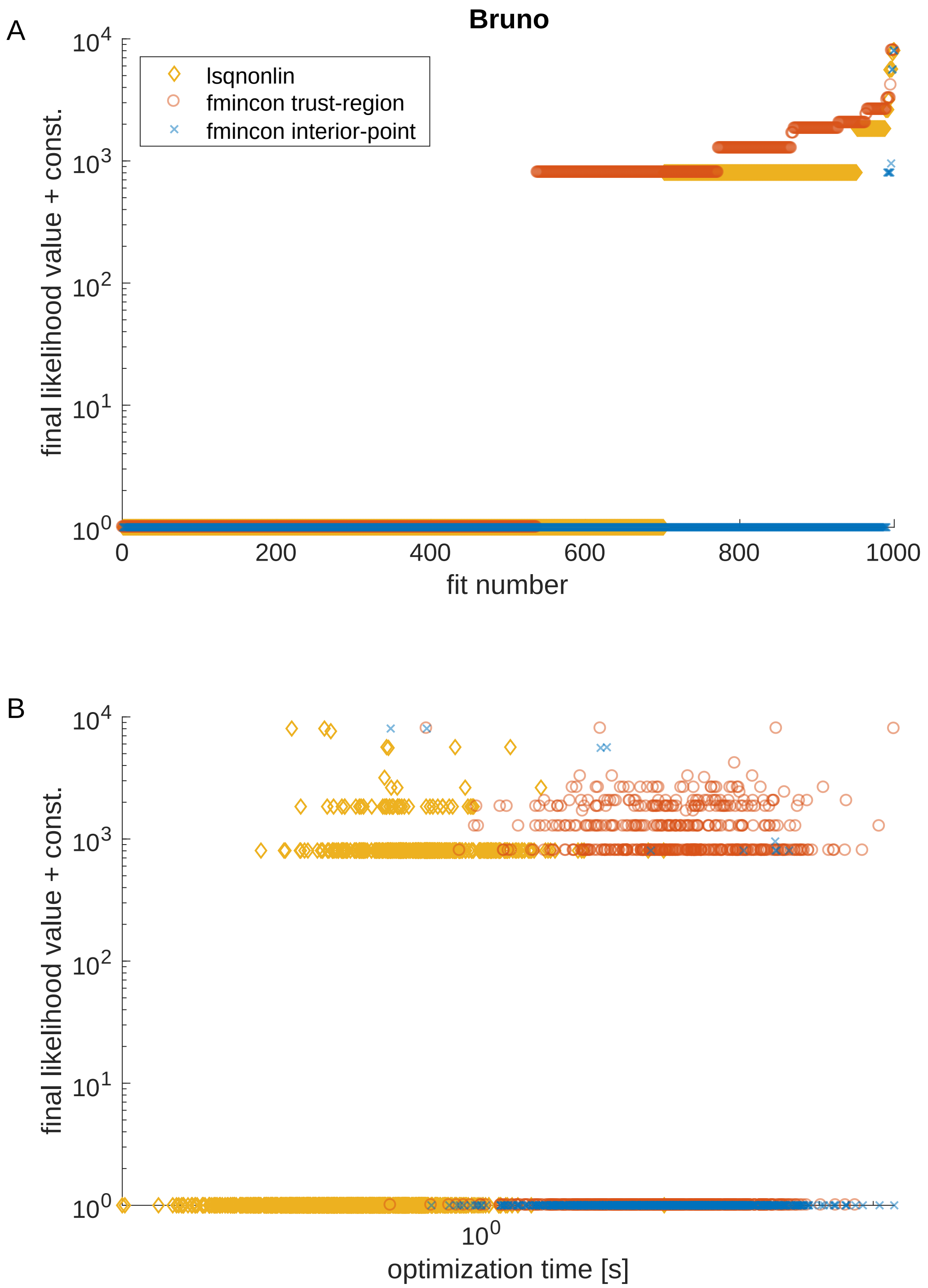

Supplementary Figure S12: (A) Waterfall plot for the Bruno benchmark model (Bruno et al., 2016b). Sorted likelihood values, with best fit value shifted to 1, are shown on the y-axis for 1000 optimization runs of different optimization algorithms, starting from equal random initial parameter guesses. (B) Scatter plot of the final likelihood values vs. required optimization time.

A

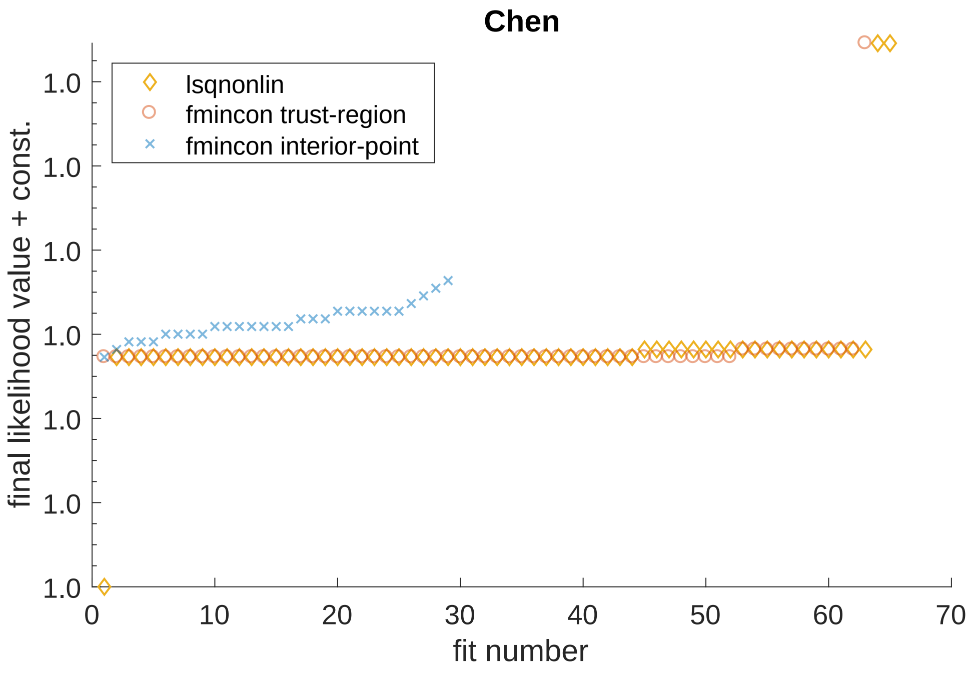

B

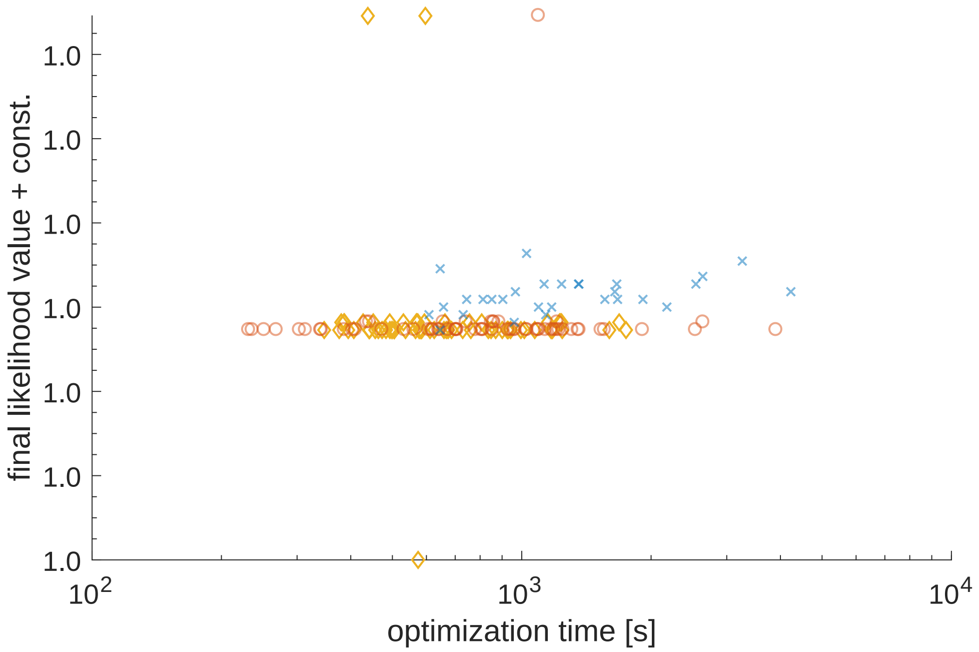

Supplementary Figure S13: (A) Waterfall plot for the Chen benchmark model (Chen et al., 2009). Sorted likelihood values, with best fit value shifted to 1, are shown on the y-axis for 1000 optimization runs of different optimization algorithms, starting from equal random initial parameter guesses. (B) Scatter plot of the final likelihood values vs. required optimization time.

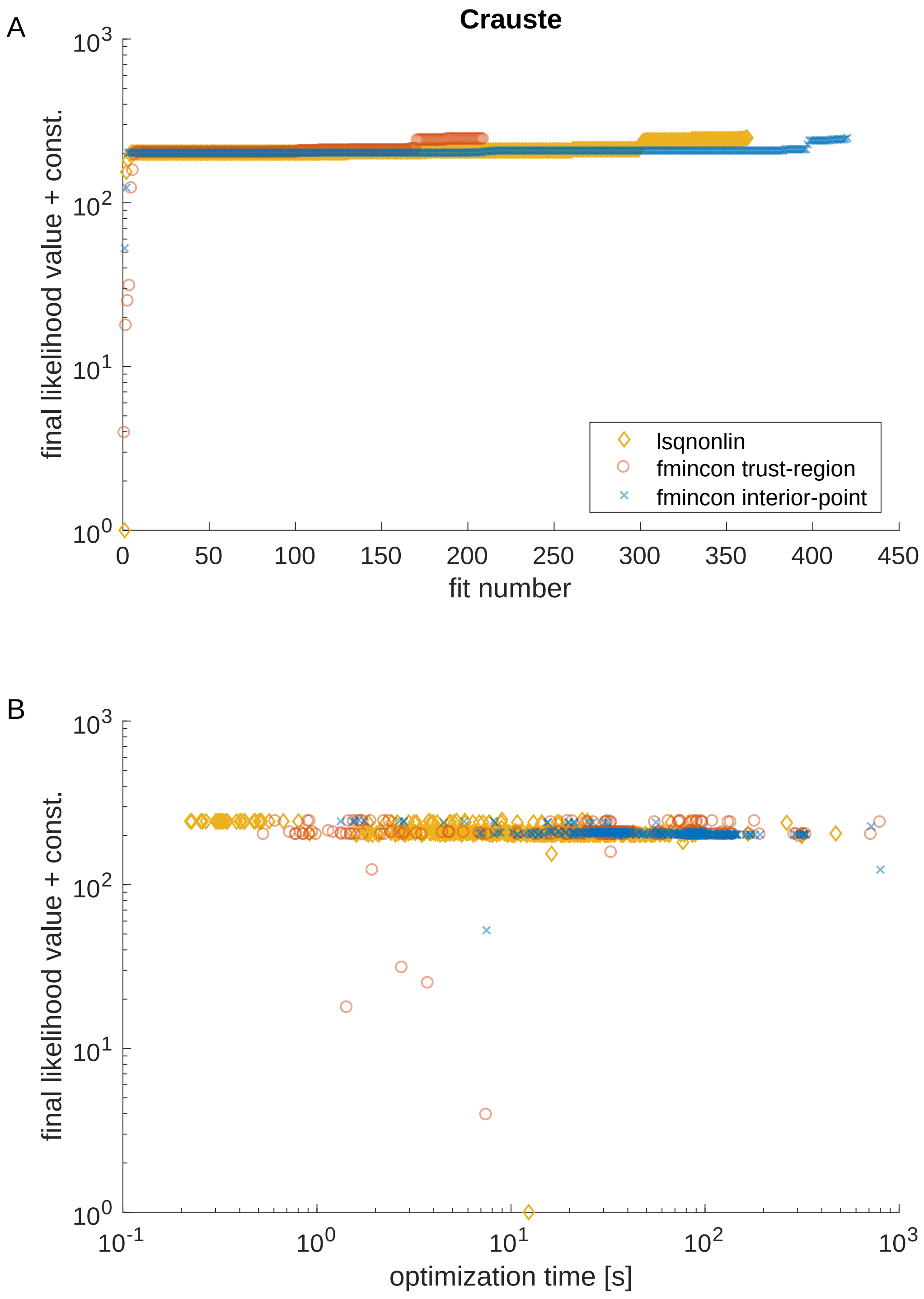

Supplementary Figure S14: (A) Waterfall plot for the Crauste benchmark model (Crauste et al., 2017). Sorted likelihood values, with best fit value shifted to 1, are shown on the y-axis for 1000 optimization runs of different optimization algorithms, starting from equal random initial parameter guesses. (B) Scatter plot of the final likelihood values vs. required optimization time.

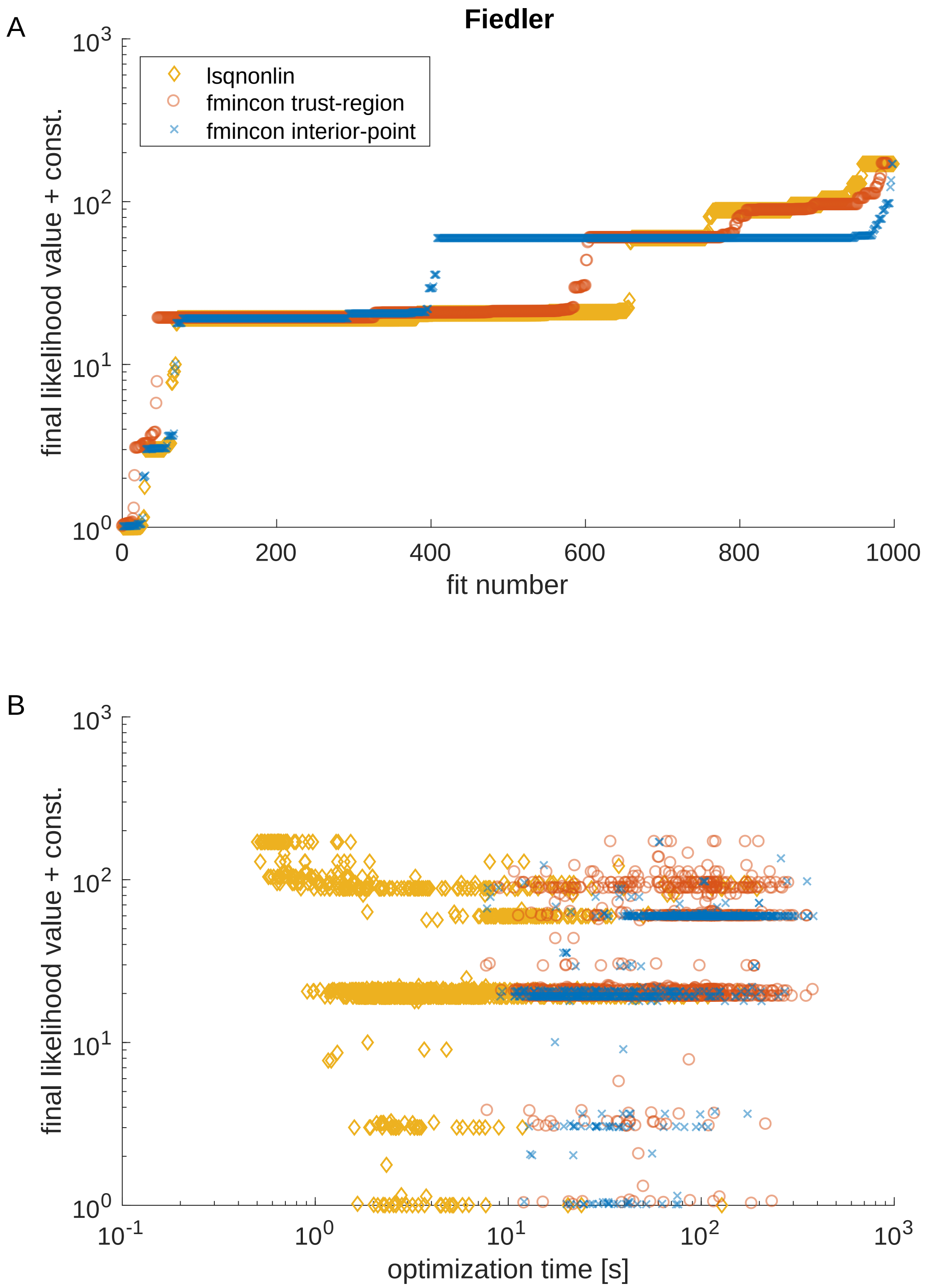

Supplementary Figure S15: (A) Waterfall plot for the Fiedler benchmark model (Fiedler et al., 2016). Sorted likelihood values, with best fit value shifted to 1, are shown on the y-axis for 1000 optimization runs of different optimization algorithms, starting from equal random initial parameter guesses. (B) Scatter plot of the final likelihood values vs. required optimization time.

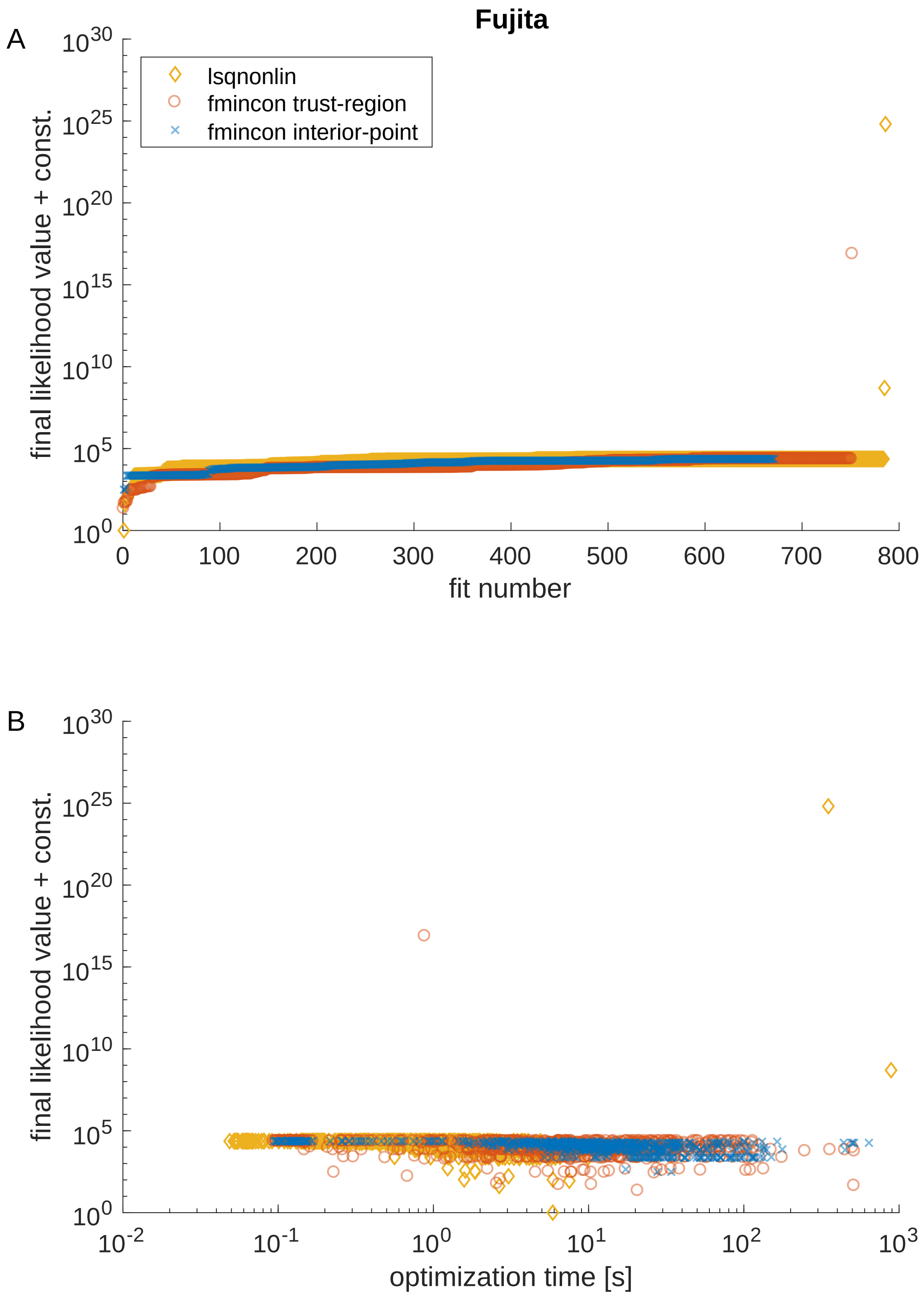

Supplementary Figure S16: (A) Waterfall plot for the Fujita benchmark model (Fujita et al., 2010). Sorted likelihood values, with best fit value shifted to 1, are shown on the y-axis for 1000 optimization runs of different optimization algorithms, starting from equal random initial parameter guesses. (B) Scatter plot of the final likelihood values vs. required optimization time.

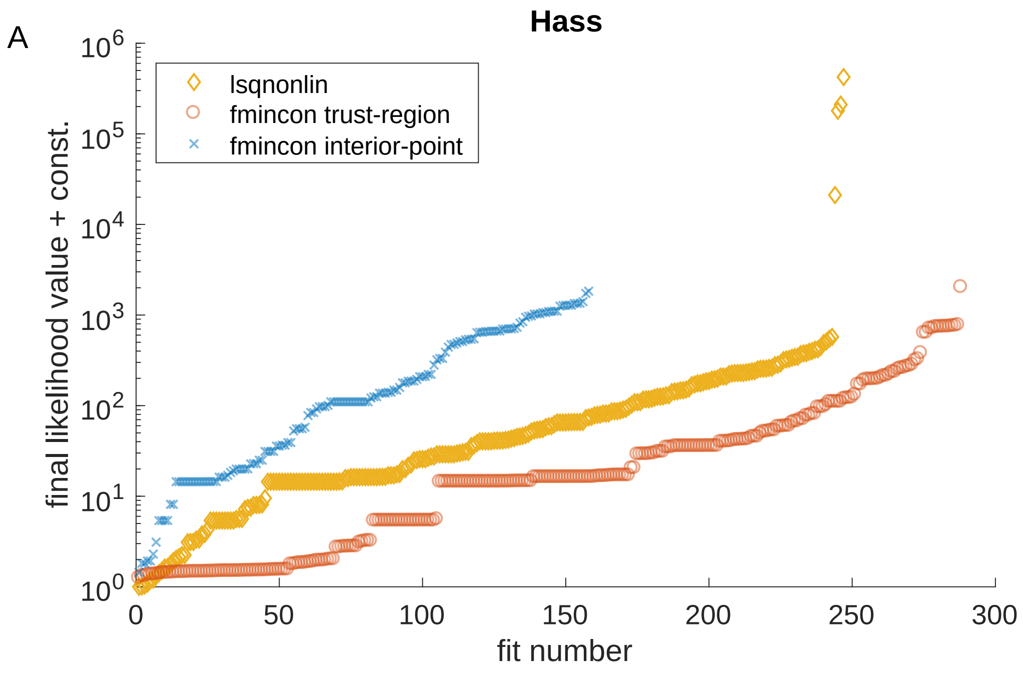

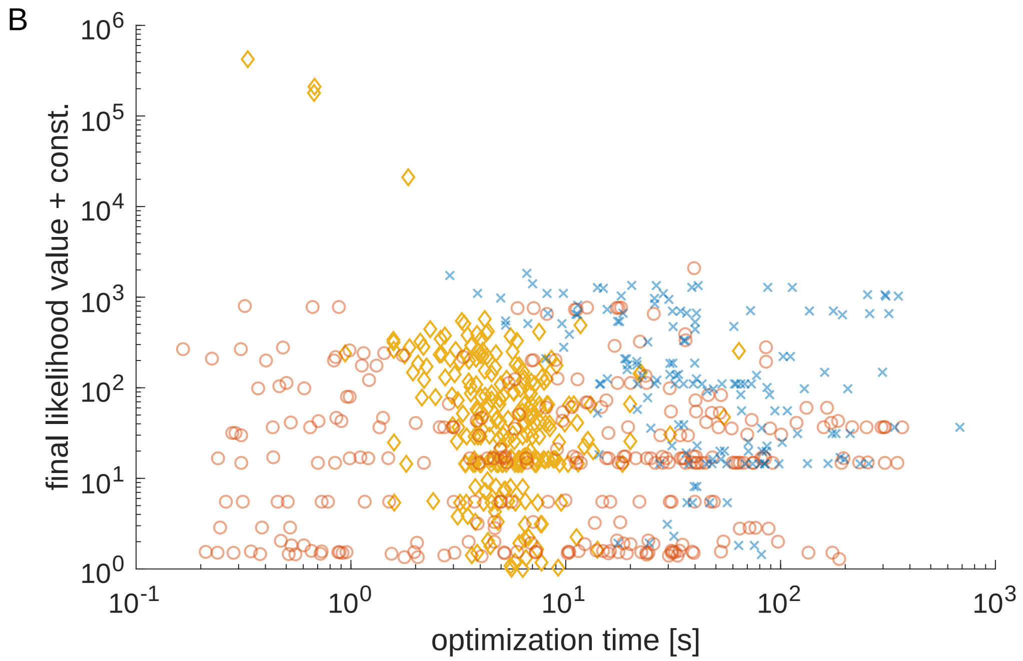

Supplementary Figure S17: (A) Waterfall plot for the Hass benchmark model (Hass et al., 2017). Sorted likelihood values, with best fit value shifted to 1, are shown on the y-axis for 1000 optimization runs of different optimization algorithms, starting from equal random initial parameter guesses. (B) Scatter plot of the final likelihood values vs. required optimization time.

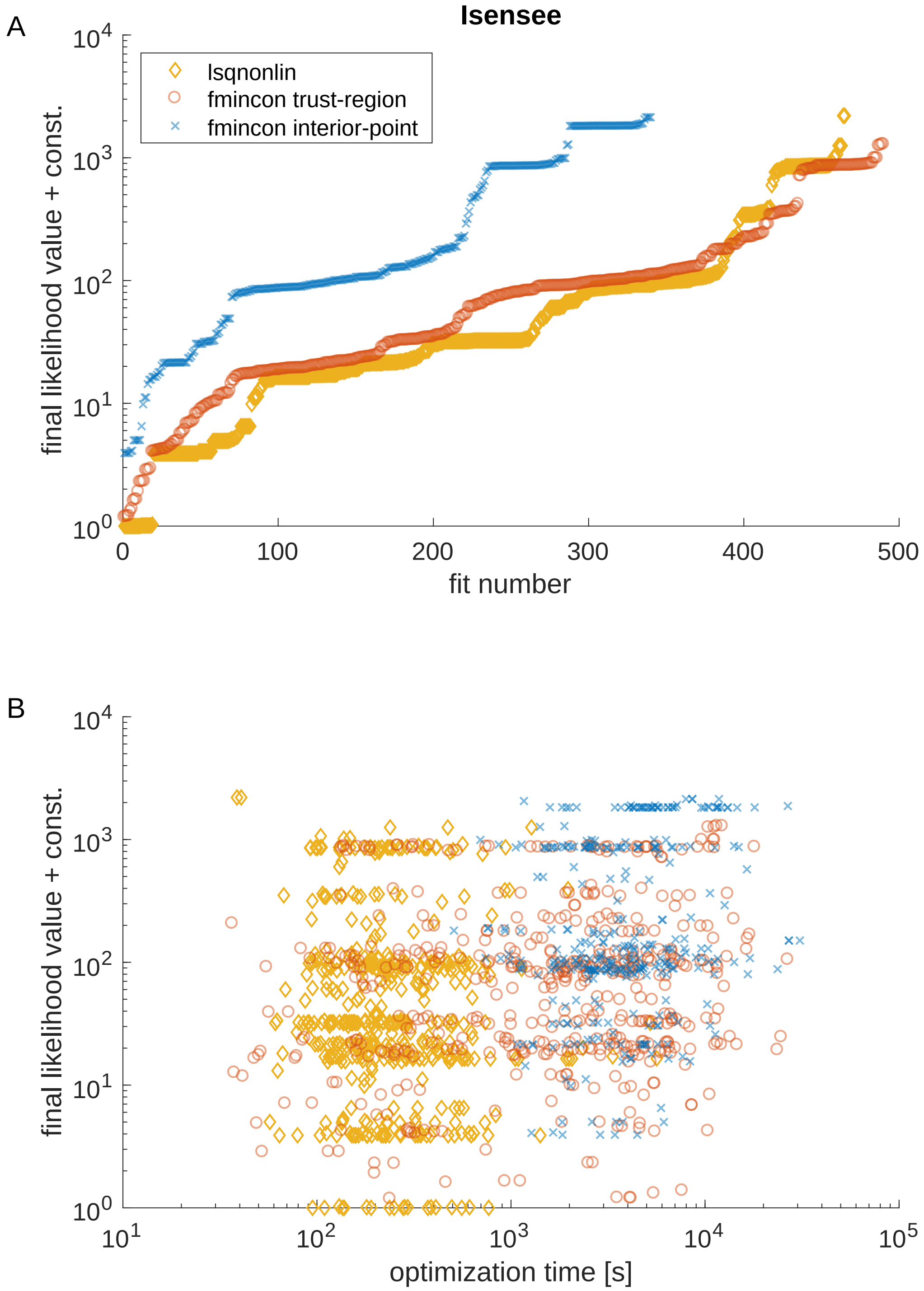

Supplementary Figure S18: (A) Waterfall plot for the Isensee benchmark model (Isensee et al., 2018). Sorted likelihood values, with best fit value shifted to 1, are shown on the y-axis for 1000 optimization runs of different optimization algorithms, starting from equal random initial parameter guesses. (B) Scatter plot of the final likelihood values vs. required optimization time.

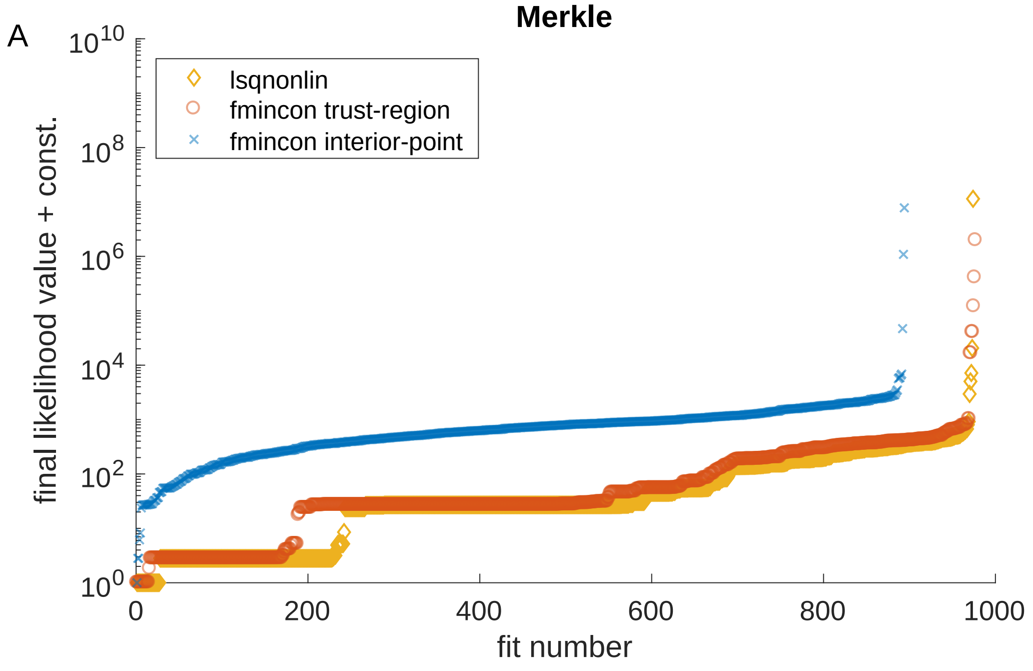

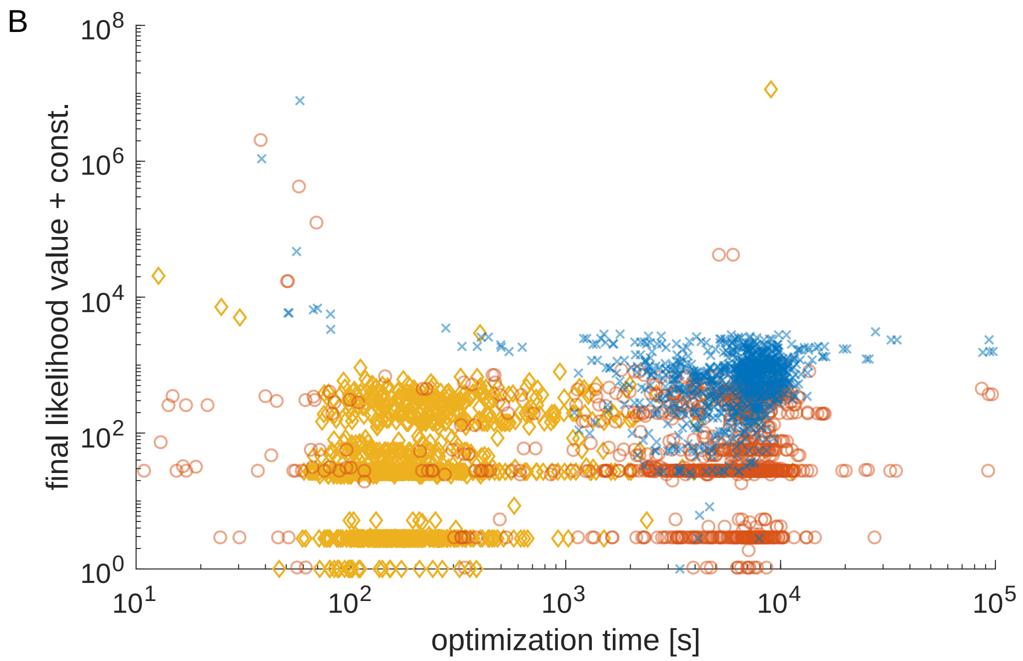

Supplementary Figure S19: (A) Waterfall plot for the Merkle benchmark model (Merkle et al., 2016). Sorted likelihood values, with best fit value shifted to 1, are shown on the y-axis for 1000 optimization runs of different optimization algorithms, starting from equal random initial parameter guesses. (B) Scatter plot of the final likelihood values vs. required optimization time.

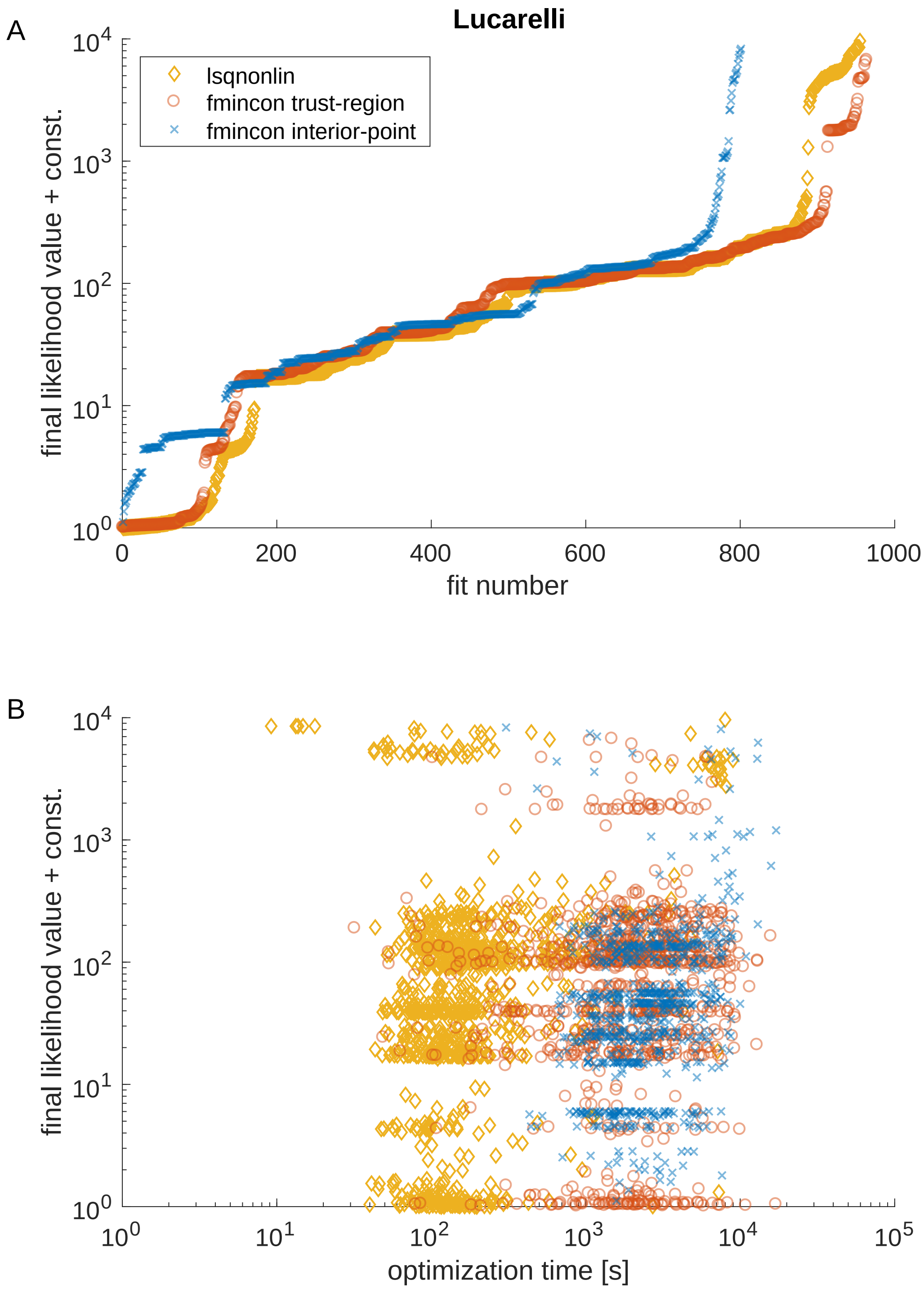

Supplementary Figure S20: (A) Waterfall plot for the Lucarelli benchmark model (Lucarelli et al., 2018). Sorted likelihood values, with best fit value shifted to 1, are shown on the y-axis for 1000 optimization runs of different optimization algorithms, starting from equal random initial parameter guesses. (B) Scatter plot of the final likelihood values vs. required optimization time.

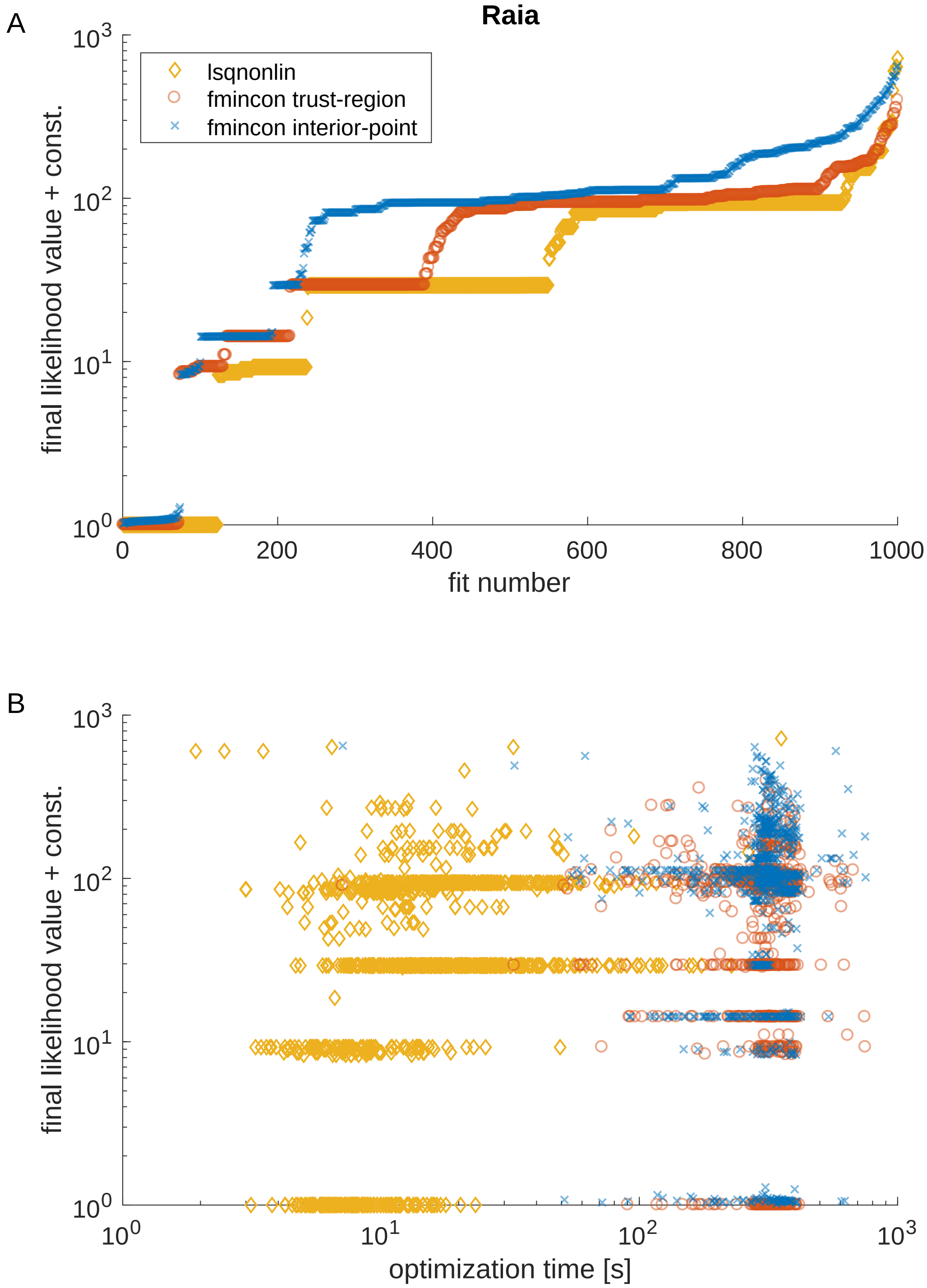

Supplementary Figure S21: (A) Waterfall plot for the Raia benchmark model (Raia et al., 2011). Sorted likelihood values, with best fit value shifted to 1, are shown on the y-axis for 1000 optimization runs of different optimization algorithms, starting from equal random initial parameter guesses. (B) Scatter plot of the final likelihood values vs. required optimization time.

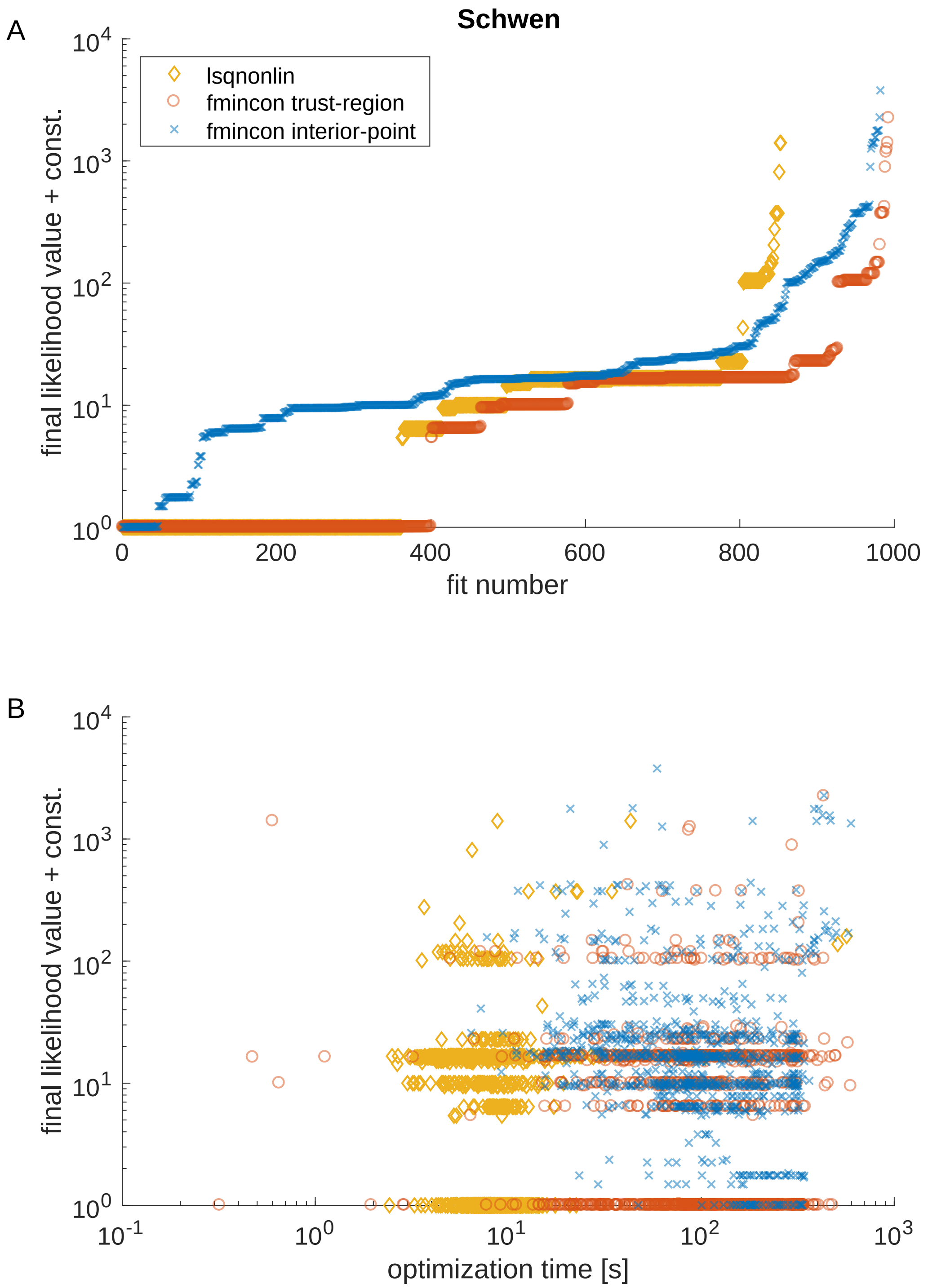

Supplementary Figure S22: (A) Waterfall plot for the Schwen benchmark model (Schwen et al., 2015). Sorted likelihood values, with best fit value shifted to 1, are shown on the y-axis for 1000 optimization runs of different optimization algorithms, starting from equal random initial parameter guesses. (B) Scatter plot of the final likelihood values vs. required optimization time.

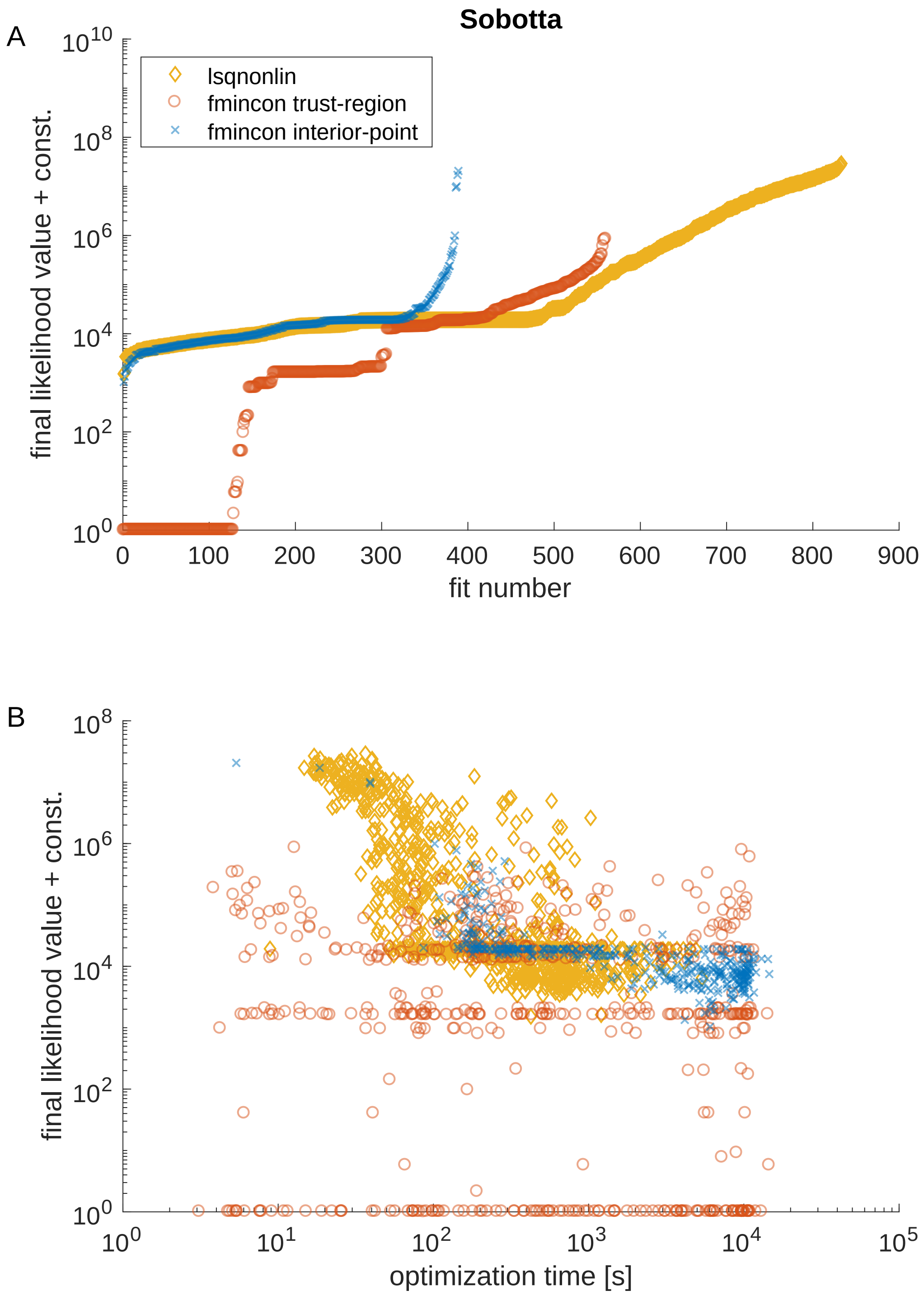

Supplementary Figure S23: (A) Waterfall plot for the Sobotta benchmark model (Sobotta et al., 2017). Sorted likelihood values, with best fit value shifted to 1, are shown on the y-axis for 1000 optimization runs of different optimization algorithms, starting from equal random initial parameter guesses. (B) Scatter plot of the final likelihood values vs. required optimization time.

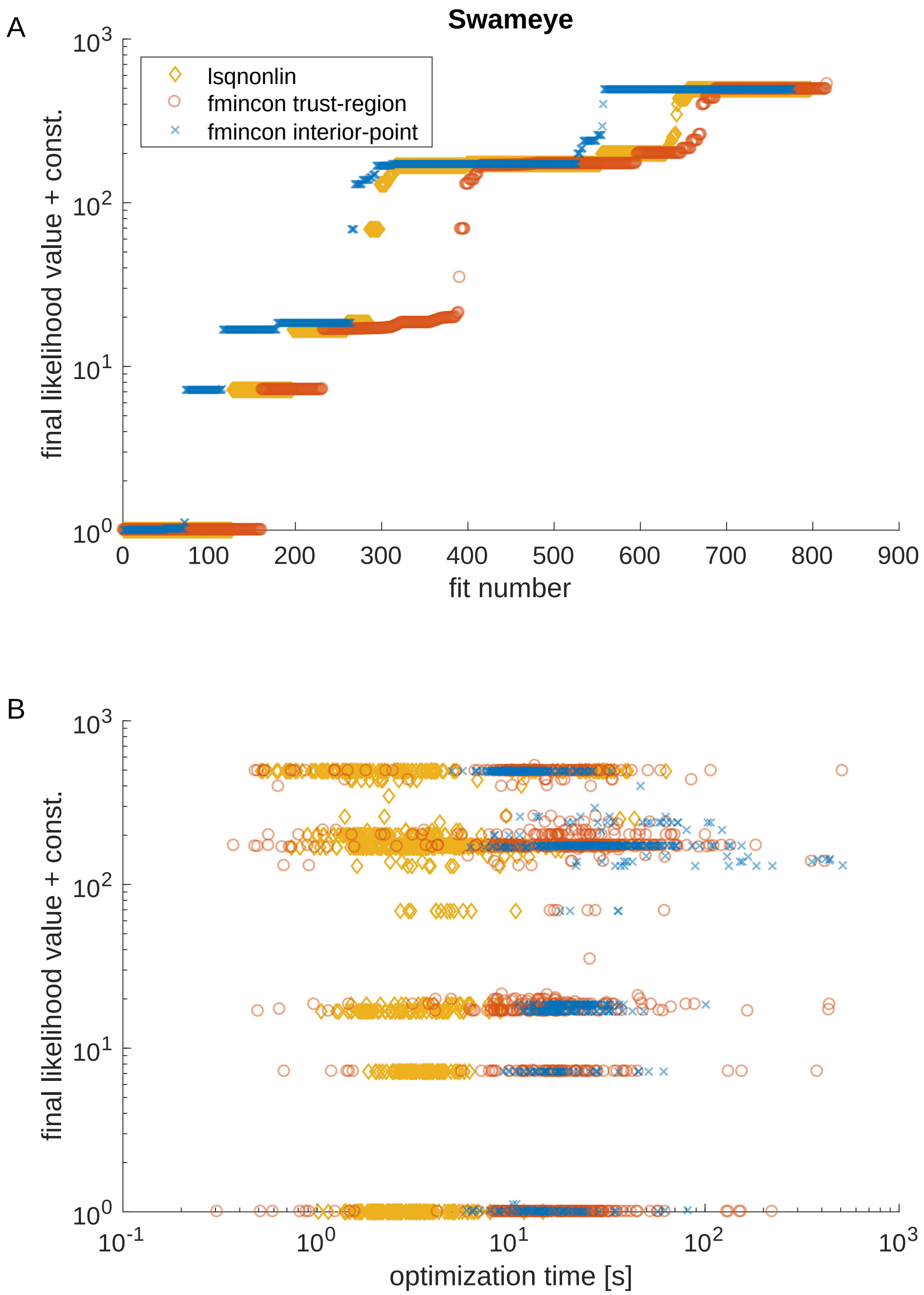

Supplementary Figure S24: (A) Waterfall plot for the Swameye benchmark model (Swameye et al., 2003). Sorted likelihood values, with best fit value shifted to 1, are shown on the y-axis for 1000 optimization runs of different optimization algorithms, starting from equal random initial parameter guesses. (B) Scatter plot of the final likelihood values vs. required optimization time.

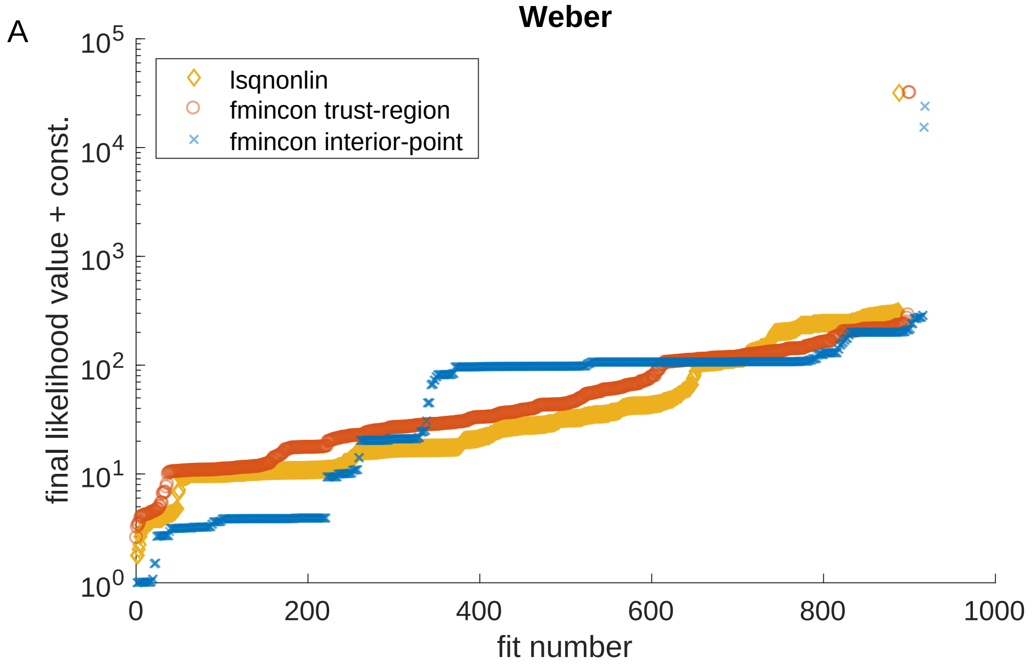

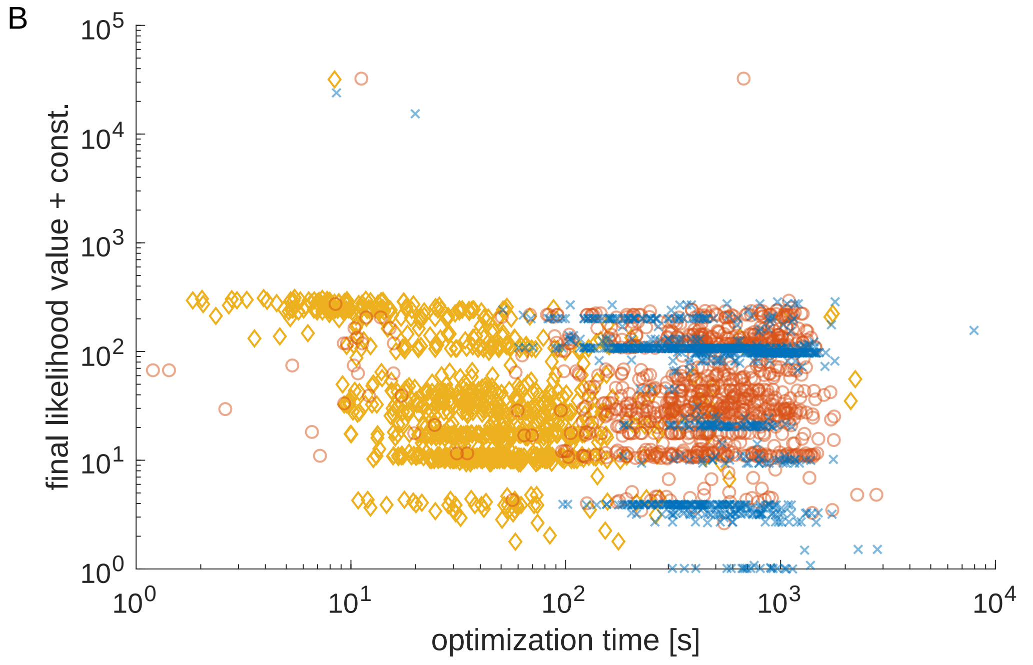

Supplementary Figure S25: (A) Waterfall plot for the Weber benchmark model (Weber et al., 2015). Sorted likelihood values, with best fit value shifted to 1, are shown on the y-axis for 1000 optimization runs of different optimization algorithms, starting from equal random initial parameter guesses. (B) Scatter plot of the final likelihood values vs. required optimization time.

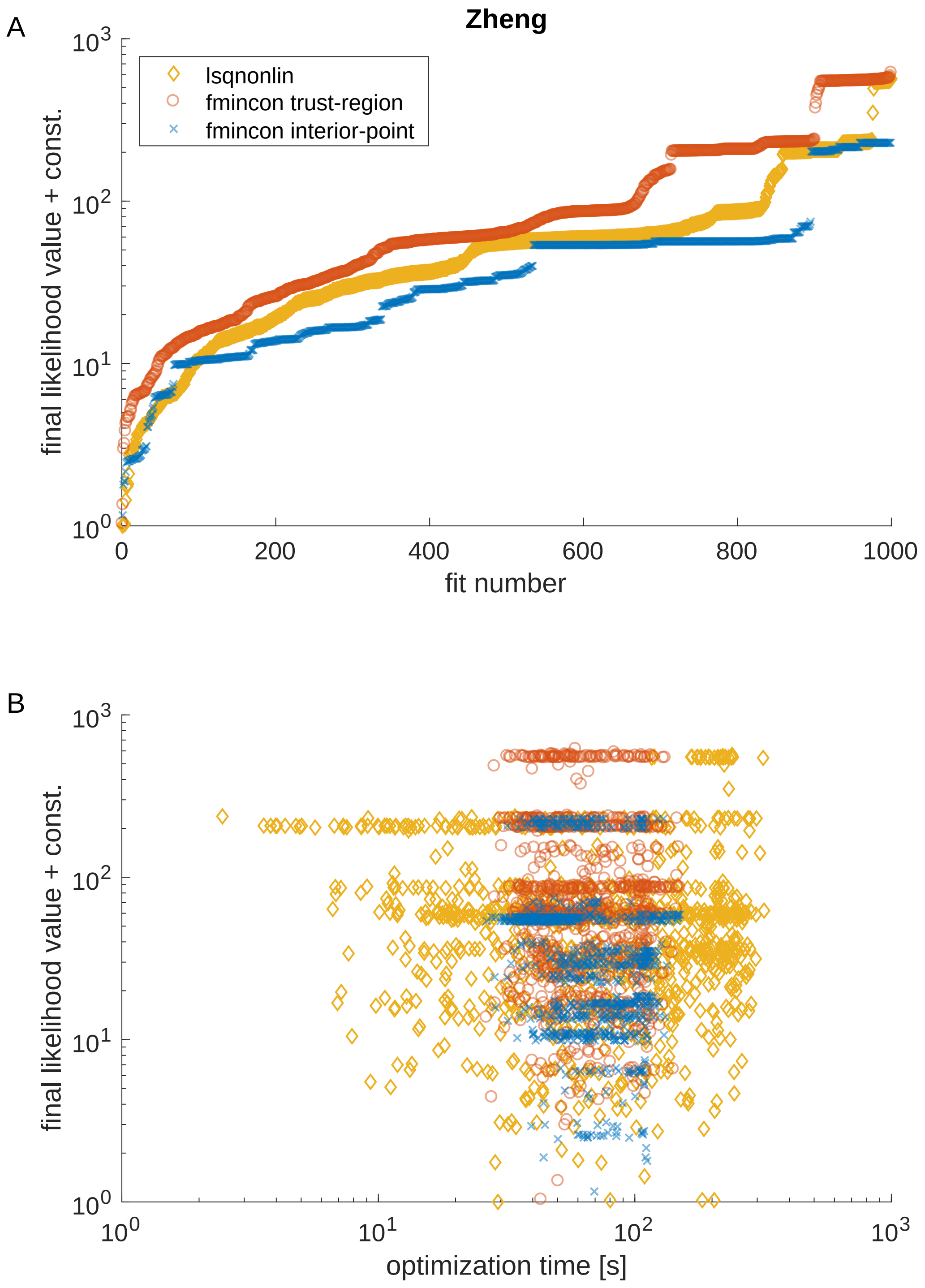

Supplementary Figure S26: (A) Waterfall plot for the Zheng benchmark model (Zheng et al., 2012). Sorted likelihood values, with best fit value shifted to 1, are shown on the y-axis for 1000 optimization runs of different optimization algorithms, starting from equal random initial parameter guesses. (B) Scatter plot of the final likelihood values vs. required optimization time.

6 Example of turnover reaction

The simple turnover reaction model used to illustrate the level sets shown in the main manuscript in Fig. 2B is depicted in Fig. S27A. This system corresponds to the ODE

$$\dot{x}(t,\theta) = \theta_1 - \theta_3\, x(t,\theta) \tag{3}$$

with initial condition $x(0) = 0$ and observation function

$$g(x(t,\theta),\theta) = \theta_2\, x(t,\theta), \tag{4}$$

which has the solution

$$g(x(t,\theta),\theta) = \frac{s\,\theta_1}{\theta_3}(1 - e^{-\theta_3 t}). \tag{5}$$

The degradation rate $\theta_3 = 1$ was assumed to be known and the scaling factor θ_2 and synthesis rate θ_1 were estimated from the data using the objective function

$$J(\theta) = \sum_{i=1}^{10} \left(g(x(t_i,\theta),\theta) - y_i\right)^2 + \sum_{j=1}^{2} \left(\frac{\theta_j - 1}{10}\right)^2,$$

with a Gaussian prior with mean=1 and standard deviation equals to 10 for the parameters θ_1 and θ_2. The data y_i was generated with additive independent normally distributed noise with $\sigma = 0.05$ around the true trajectory for $\theta = (1,1,1)$.

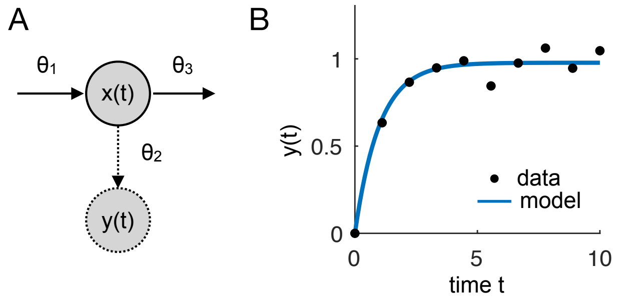

Supplementary Figure S27: (A) Illustration of the simple turnover reaction. (B) Simulated measurement data and model fit using the optimal parameters.

7 Impact of parameter transformation of the convexity of the objective function

The impact of a log-transformation of model parameters on the convexity of the likelihood landscape was assessed for investigating a potential explanation for the performance benefits of numerical optimization at log scale.

A function is convex if for all points $\theta^{(1)}, \theta^{(2)}$, the linear interpolation

$$L(\alpha) = \alpha J(\theta^{(1)}) + (1-\alpha) J(\theta^{(2)}) \tag{6}$$

is greater than $J(\alpha\theta^{(1)} + (1-\alpha)\theta^{(2)})$, i.e.

$$J(\alpha\theta^{(1)} + (1-\alpha)\theta^{(2)}) \leq \alpha J(\theta^{(1)}) + (1-\alpha) \ \ , \forall \alpha \ . \tag{7}$$

An equivalent criterion for twice differentiable function $J(\theta)$ is that its Hessian is positive definite:

$$\forall \theta' : \quad \left. \frac{d^2 J}{d\theta^2} \right|_{\theta=\theta'} \geq 0. \tag{8}$$

Since derivatives (8) are difficult to be calculated for ODE models, e.g. because numerical integration errors leads to numerically instable finite differences, we employ definition (6) for evaluation of convexity. Because numerical methods only evaluate the objective function at discrete points in the parameter space we randomly draw a point for evaluating (7).

Firstly, we sampled 1000 random parameter sets $\theta^{(1)} \in \Omega$. Secondly, we sampled a parameter set $\theta^{(2)} \in \Omega$ with $||\theta^{(2)} - \theta^{(1)}|| = 1$ was drawn. Thirdly, we sampled a random location on the connecting line, $\alpha \sim \mathcal{U}(0,1)$. Finally, we checked if (7) holds.

While a function is either convex or not, we interpret the faction of samples for which (7) does hold as the degree of convexity. Alternatively, one might consider the fraction for which (7) does not hold as the degree of non-convexity. Indeed, for a function with many local optima, one would expect that (7) is usually violated.

In this study, we considered four scenarios. Parameters that were either sampled uniformly in log- or linear-scale; and the connection line was either constructed in log- or linear-scale. Default boundaries for all model parameters were -5 to 3 on a log-scale.

Detailed results of all benchmark models are shown in Fig. S28. For most of the models, sampling the parameters and connecting them in log-scale yields the highest number of samples for which (7) holds. This suggest that the objective function is more convex in log- than in linear-scale. In addition, the improved convexity in log-space was also visible when parameters were drawn in linear space.

The overall significance of convexity in parameters sampled in log-space against linear space is $p = 0.045$, tested by signed rank sum test with null hypothesis that the connecting line in log-space leads to the same proportion of convexity as the connecting line in linear space.

Since the Chen model could not be simulated for almost all sampled parameters, we excluded the model from this analysis.

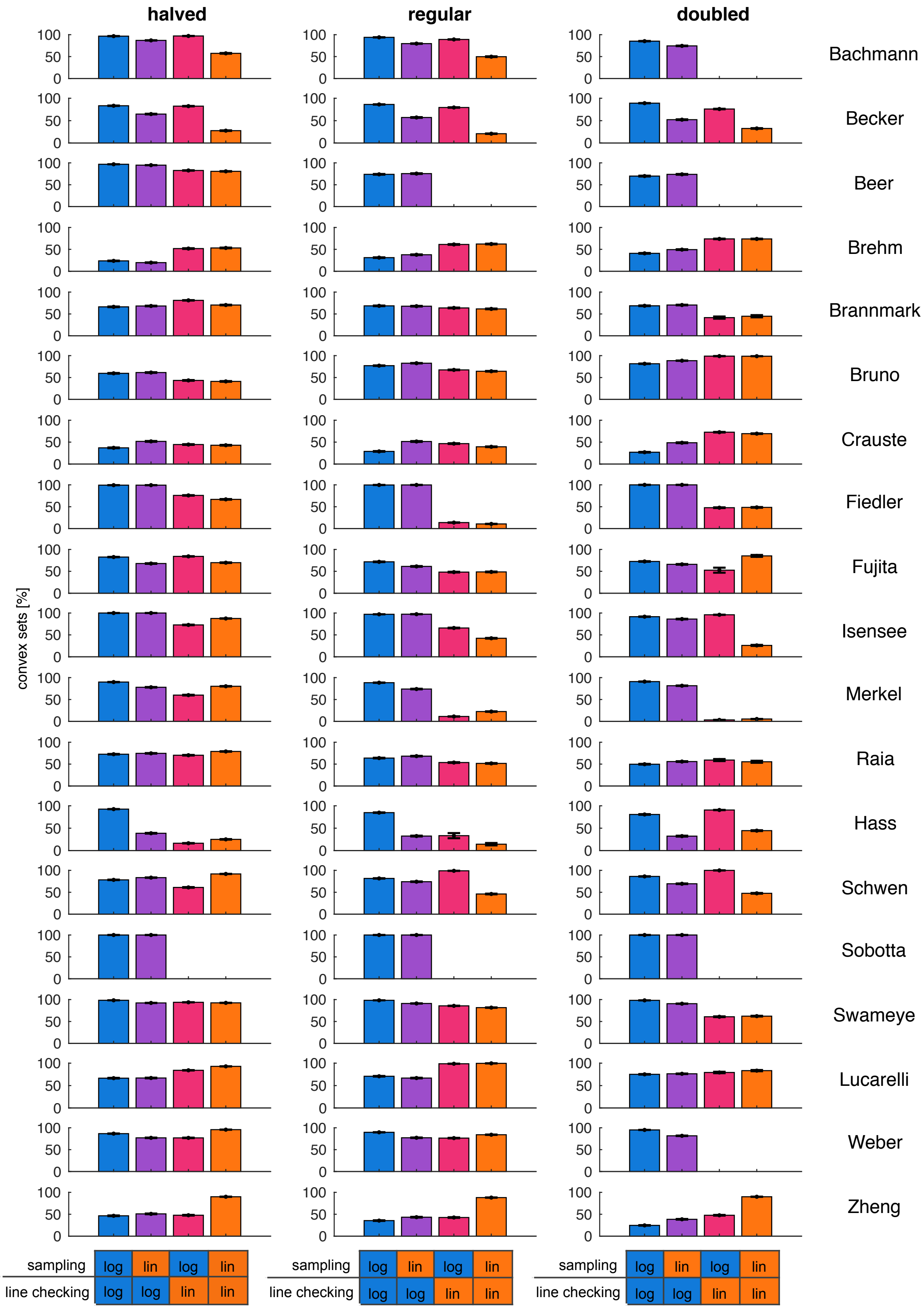

Supplementary Figure S28: **Measure of convexity for different setups.** Both sampling of the two parameter sets and the line connecting it were drawn either in log or linear space. The convexity of this set is shown for each of the 20 benchmark models as percentage. Each row is for one model, and the columns represent different parameter boundaries. The left column shows the results for halved parameters boundaries, the middle for the regular boundaries and the right for doubled boundaries.

8 Number of converged local optimization runs

The performance metric used in this manuscript accounts for the average computation time of a local optimizer and the percentage of converged runs. To assess whether there are differences for the latter between lsqnonlin, fmincon with algorithm-option *trust-region-reflective* and fmincon with algorithm-option *interior-point*, we evaluated the percentage of converged starts for all methods and benchmark problems. Our analysis revealed that lsqnonlin mostly outperforms fmincon with the algorithm-option *trust-region-reflective* and *interior-point* (Fig. S29).

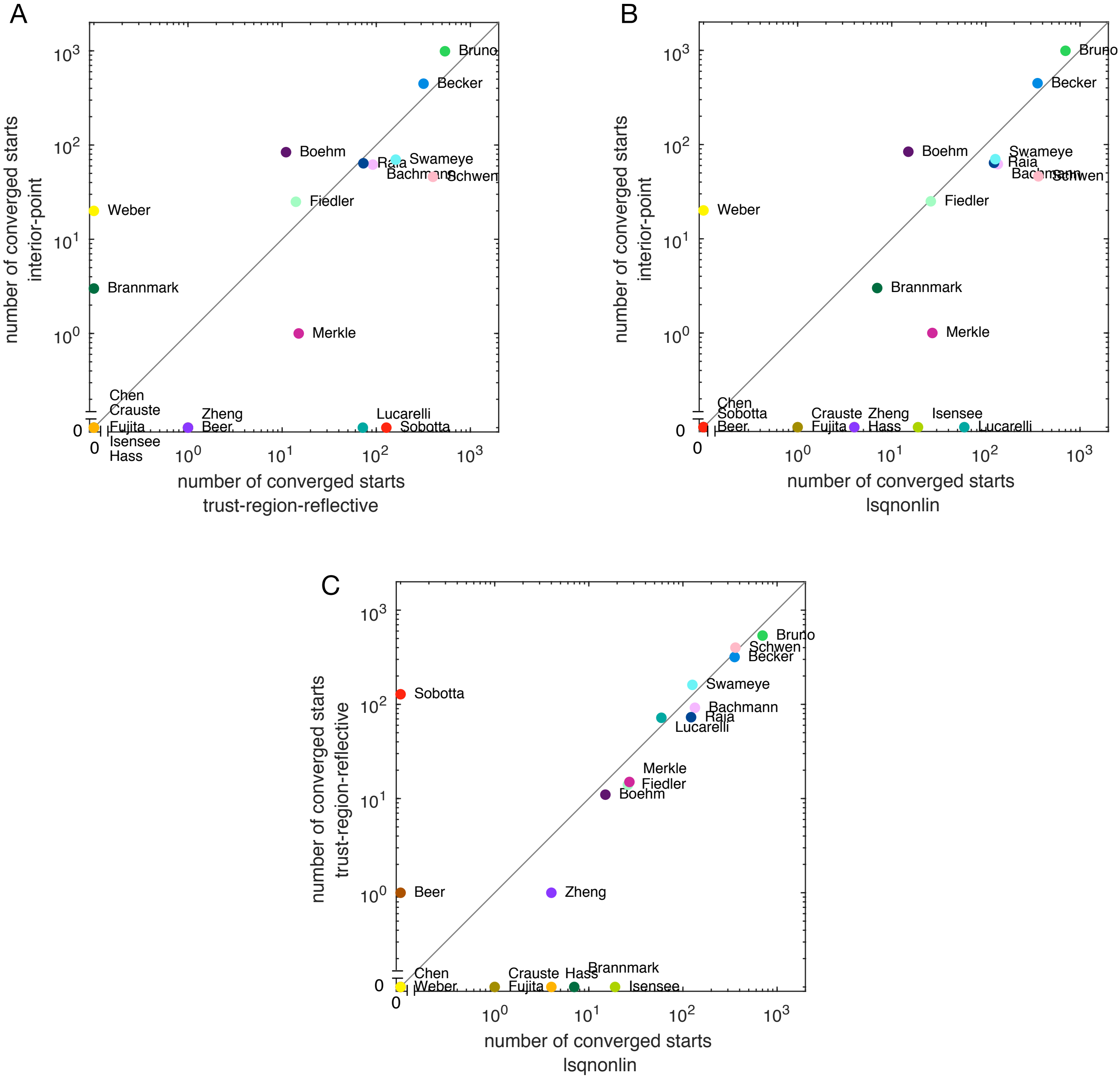

Supplementary Figure S29: **Comparison of convergence for the optimizers.** Scatter plot of the number of converged starts for the interior-point algorithm, trust-region-reflective algorithm and lsqnonlin algorithm for a threshold of 0.1.

9 Influence of threshold for defining convergence

In the main manuscript, we showed the results for which optimization runs were considered to be converged when the objective function value differs at most by 10^{-1} from the globally best objective function value found across all runs for the given benchmark problem. The performance metric was then defined as average number of converged starts per minute (see Villaverde et al. (2018)), whereby models where only one single fit marks the globally best objective function value were omitted. We assessed the influence of this threshold on the results by evaluating additional thresholds for the analysis of the log-transformation (Fig. S30) as well as for the comparison of trust-region-reflective and interior-point (Fig. S31). The results qualitatively coincide for all thresholds.

We note that for all evaluations absolute differences between values of the log-likelihood functions was used. As (i) uncertainty analysis such as profile likelihoods as well as (ii) model selection criteria consider absolute differences, the optimizers need to achieve a small absolute error. Small relative errors are insufficient despite the fact that the optimal log-likelihood values for different models are on different scales.

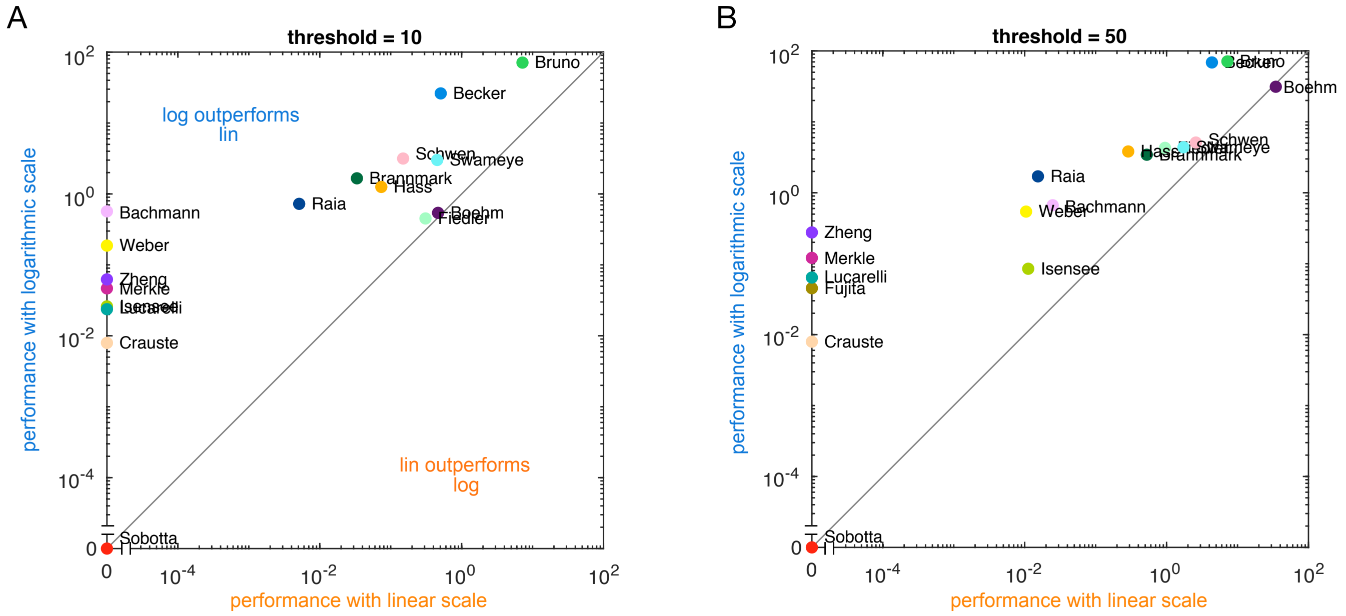

Supplementary Figure S30: **Comparison of optimizer performance in linear and log scale for different thresholds used to define convergence.** Performance of the multi-start local optimization scheme using the MATLAB optimizer lsqnonlin for: sampling in log scale and optimization in linear scale; and sampling and optimization in logarithmic scale for thresholds (A) 10 and (B) 50.

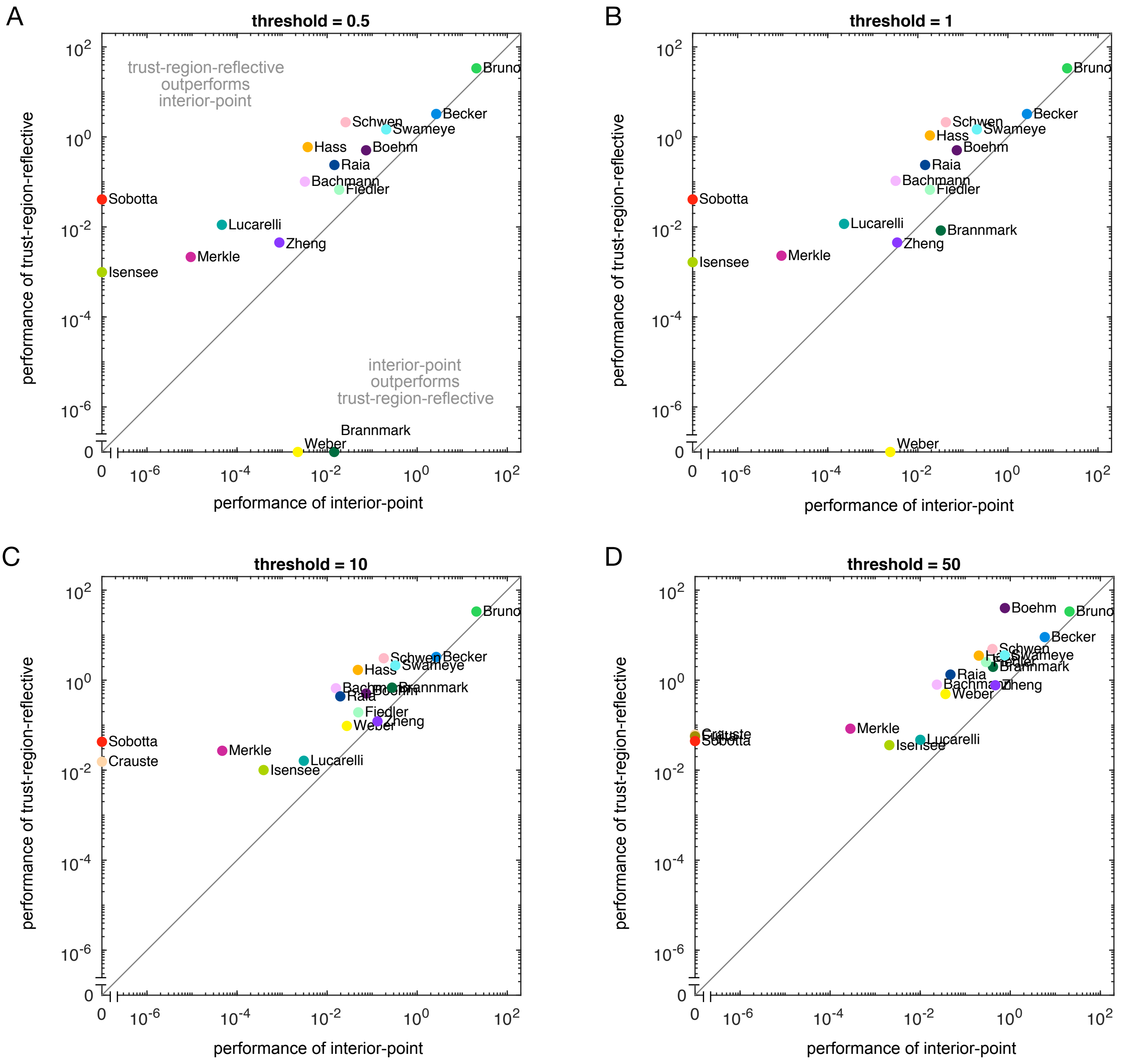

Supplementary Figure S31: **Comparison of optimizer performance for different thresholds used to define convergence.** Scatter plot of the average number of converged starts per minute for the interior-point algorithm vs. trust-region-reflective algorithm for thresholds (A) 0.5, (B) 1, (C) 10 and (D) 50.

10 Correlation of number of function evaluations and iterations

For the considered optimizers, we analyzed the correlation between function evaluations and iterations of the algorithms. For all optimizers, we observe a high correlation. The result for fmincon interior-point are shown in Figure S32. For the other optimizers, it is always one and two more function evaluations than iterations for lsqnonlin and fmincon trust-region reflective, respectively. This yields a correlation of 1.

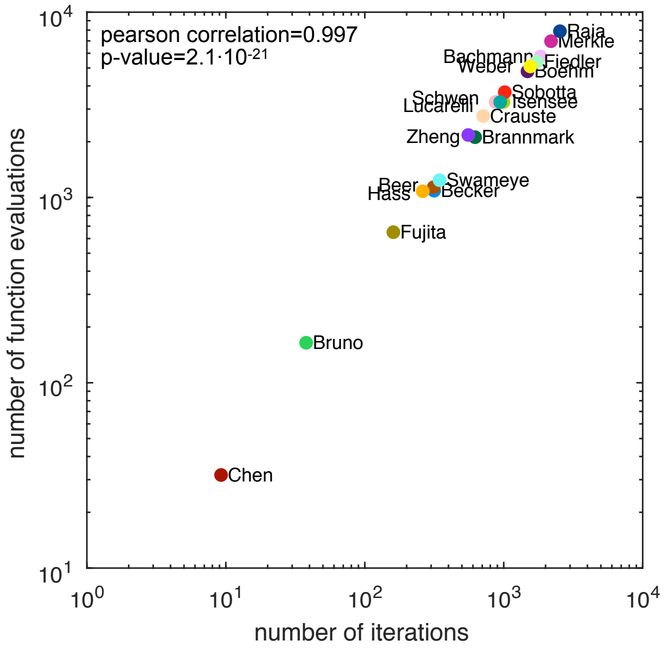

Supplementary Figure S32: The mean number of function evaluations and iterations are highly correlated. The results are shown for fmincon algorithm interior-point.

11 Optimization results on simulated data

We generated simulated data with the estimated noise levels and repeated the optimization using lsqnonlin. The comparison of the performance of the optimization for the simulated data and the experimental data are shown in Fig. S33A. We tested the hypothesis that optimization for simulated data works significantly better. For this we analyzed the log-performance difference between simulated and experimental data, i.e.,

$$\text{log-performance difference} := \log_2(\text{performance for simulated data}) - \log_2(\text{performance for experimental data}).$$

A one-sided Wilcoxon signed rank test revealed that this difference is greater than zero with p-value < 0.1 and a one-sided t-test gave a p-value of 0.03 (Fig. S33B). The median of the log-performance difference is 0.19, while the mean is 0.83. For the subset of non-identifiable models, these differences were even bigger (median=0.26 and mean 0.9).

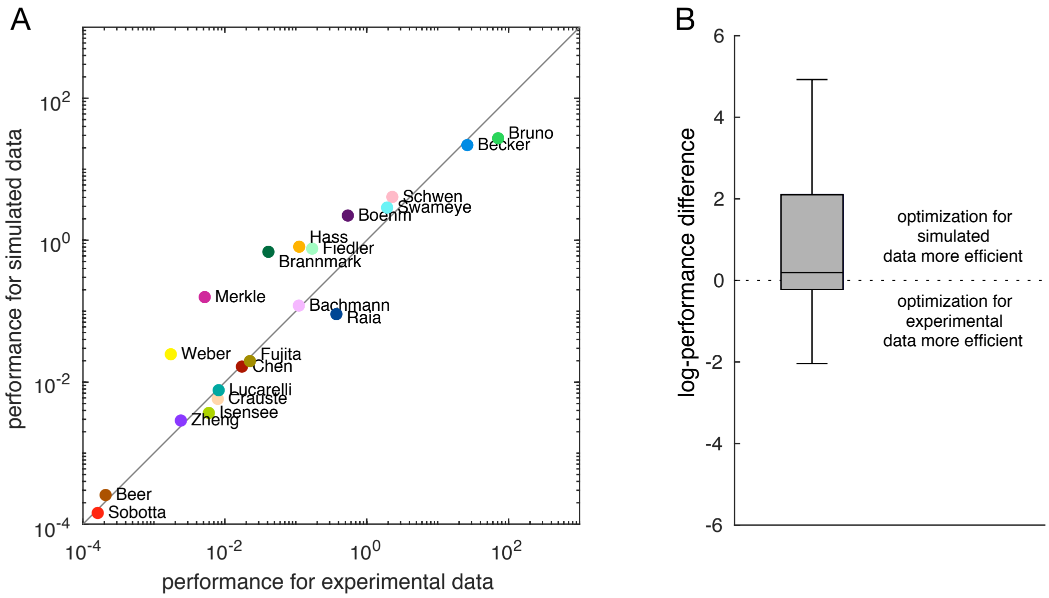

Supplementary Figure S33: Comparison of the performance for simulated and experimental data. (A) The performance is shown for simulated and experimental data for optimization with lsqnonlin. (B) Boxplot for the log-performance difference.

12 Identifiability analysis

Identifiability of the model parameters was analyzed in terms of unique parameter estimates, i.e. it has been investigated whether the minimum of the negative log-likelihood of the data is given by a unique parameter vector. Non-uniqueness might either originate from a-priori non-identifiability, i.e. from structural non-identifiability of the model equations, or from practical non-identifiability raised by limited amount of data. Moreover, the assignment to structural and practical depends on the question whether observation parameters like scalings or offsets are considered as part of the model structure, or whether non-identifiabilities related to these parameters are practical because of practical limitations for directly observing the biological system.

Uniqueness of the estimated parameters was analyzed using the *identifiability test by radial penalization (ITRP)* (Kreutz, 2018). This approach omits classification into structural or practical and has two major steps:

1. Classical parameter estimation, e.g. by minimizing the negative log-likelihood yielding estimates $\hat{\theta}$
2. Minimization of a penalized negative log-likelihood and evaluating whether the same value of the unpenalized part is obtained.

In the second step, the negative log-likelihood used as classical merit function is augmented by adding a penalty which is proportional to

$$P(\theta) = \left(\sqrt{\sum_i \left(\hat{\theta}_i - \theta_i \right)^2} - R \right)^2 . \tag{9}$$

This penalty $P(\theta)$ has radial symmetry around $\hat{\theta}$ and is minimal with $P(\theta) = 0$ on a sphere with radius R. Such a radial penalty pulls away from the first estimate $\hat{\theta}$ and thereby a new minimum is searched in arbitrary direction on the sphere with radius R. In our analysis we chose $R = 1$ on the $\log_{10}$-parameter scale as suggested in the original publication. For further details we refer to (Kreutz, 2018).

13 Analysis of sloppiness

The empirically observed *Fisher information* (Kreutz and Timmer, 2013) is the Hessian of the log-likelihood for the estimated parameters, and coincides with the Hessian of the least-squares objective function in the case of normally distributed noise. The diagonal elements $(H)_{ii}$ of this Hessian H are traditionally used for assessing parameter uncertainties and for the calculation of standard errors of estimated parameters.

For ODE models as they are applied in systems biology, it has been observed that the eigenvalues of the Hessian of the least-squares objective function are spread over several orders of magnitude, even if the parameters are considered at the log-scale (Gutenkunst et al., 2007). This characteristic has been termed as *sloppiness* (Waterfall et al., 2006). In (Gutenkunst et al., 2007), sloppiness was observed for every ODE model evaluated from a collection of 17 systems biology models from the literature and was therefore claimed as a universal property of such application models which hampers parameter estimation (Scheff et al., 2013; Transtrum et al., 2010).

In Tönsing et al. (2014), it has been shown that the origin of sloppiness partly originates from the eigenvalue calculation, and predominantly from the fact that correlated observations are provided by time-course observations of a subset of the underlying compounds. Moreover, it was argued (Apgar et al., 2010; Chis et al., 2016; Tönsing et al., 2014) that sloppiness of the eigenvalues of the Hessian does not automatically prevent identifiability of parameters and that parameter uncertainties are a matter of the experimental design and should not be interpreted as an universal characteristic.

In Gutenkunst et al. (2007), the approximation of a Hessian H_{sim},

$$(H_{\text{sim}})_{ij} = \frac{\partial^2}{\partial\theta_i\partial\theta_j}\chi^2_{\text{sim}}(\theta)|_{\theta=\hat{\theta}} \approx \frac{\partial}{\partial\theta_i}\chi^2_{\text{sim}}(\theta)|_{\theta=\hat{\theta}} \cdot \frac{\partial}{\partial\theta_j}\chi^2_{\text{sim}}(\theta)|_{\theta=\hat{\theta}} \tag{10}$$

with

$$\chi^2_{\text{sim}}(\theta) = \sum \left(\frac{g_i(\theta) - g_i(\hat{\theta})}{s_i}\right)^2 \tag{11}$$

with "normalization constants" s_i, was calculated as the second derivatives of the model predictions g_i with respect to the logarithms of the parameters are computationally challenging. Because our benchmark models provide experimental data, it enables the evaluation of sloppiness using derivatives

$$(H)_{ij} = \frac{\partial^2}{\partial\theta_i\partial\theta_j}\log(\mathcal{L}(\theta))|_{\theta=\hat{\theta}} \approx \frac{\partial}{\partial\theta_i}\log(\mathcal{L}(\theta))|_{\theta=\hat{\theta}}\frac{\partial}{\partial\theta_j}\log(\mathcal{L}(\theta))|_{\theta=\hat{\theta}} \tag{12}$$

of log-likelihood $\log(\mathcal{L})$, i.e. evaluation of exactly the same objective function as used for estimation of the parameters.

In the setting of estimation from experimental data, the set of unknown parameters comprises parameters of the ODEs and, in contrast to the simulation-based setting investigated in Gutenkunst et al. (2007), also involves unknown parameters of the observation functions and of the error model. Our benchmark collection allows the comprehensive evaluation of sloppiness by consideration of dynamic parameters, observation- and error parameters as they occur in realistic applications.

As shown in the main manuscript, we found that in agreement with earlier observations, the models exhibit large spreads of the eigenvalue spectra. For 19 models, the spectra cover more than six orders of magnitude

and are therefore sloppy according to the traditional definition. However, the model published in (Bruno et al., 2016a) is non-sloppy which emphasizes that appearance of sloppiness is a matter of the experimental design. Many models show a spread over more than 15 orders. This is only partly explained by non-identifiability. In general, the Hessian becomes singular in the case of non-unique estimates, e.g. raised by structural non-identifiability. Then, there are eigenvalues equals to zero. As written in the Figure caption, such eigenvalues were set to 10^{-20} for plotting the spectra on the log-scale.

References

Apgar, J. F., Witmer, D. K., White, F. M., and Tidor, B. (2010). Sloppy models, parameter uncertainty, and the role of experimental design. *Mol. BioSyst.*, 6:1890–1900.

Bachmann, J., Raue, A., Schilling, M., Böhm, M. E., Kreutz, C., Kaschek, D., Busch, H., Gretz, N., Lehmann, W. D., Timmer, J., and Klingmüller, U. (2011). Division of labor by dual feedback regulators controls JAK2/STAT5 signaling over broad ligand range. *Mol. Syst. Biol.*, 7:516.

Becker, V., Schilling, M., Bachmann, J., Baumann, U., Raue, A., Maiwald, T., Timmer, J., and Klingmüller, U. (2010). Covering a broad dynamic range: information processing at the erythropoietin receptor. *Science*, 328(5984):1404–1408.

Beer, R., Herbst, K., Ignatiadis, N., Kats, I., Adlung, L., Meyer, H., Niopek, D., Christiansen, T., Georgi, F., Kurzawa, N., Meichsner, J., Rabe, S., Riedel, A., Sachs, J., Schessner, J., Schmidt, F., Walch, P., Niopek, K., Heinemann, T., Eils, R., and Di Ventura, B. (2014). Creating functional engineered variants of the single-module non-ribosomal peptide synthetase IndC by T domain exchange. *Mol. Biosyst*, 10(7):1709–1718.

Boehm, M. E., Adlung, L., Schilling, M., Roth, S., Klingmüller, U., and Lehmann, W. D. (2014). Identification of isoform-specific dynamics in phosphorylation-dependent STAT5 dimerization by quantitative mass spectrometry and mathematical modeling. *J. Proteome Res.*, 13(12):5685–5694.

Brännmark, C., Palmer, R., Glad, S. T., Cedersund, G., and Strålfors, P. (2010). Mass and information feedbacks through receptor endocytosis govern insulin signaling as revealed using a parameter-free modeling framework. *J. Biol. Chem.*, 285(26):20171–20179.

Bruno, M., Koschmieder, J., Wuest, F., Schaub, P., Fehling-Kaschek, M., Timmer, J., Beyer, P., and Al-Babili, S. (2016a). Enzymatic study on AtCCD4 and AtCCD7 and their potential in forming acyclic regulatory metabolites. *J. Exp. Biol.*, 67:5993–6005.

Bruno, M., Koschmieder, J., Wuest, F., Schaub, P., Fehling-Kaschek, M., Timmer, J., Beyer, P., and Al-Babili, S. (2016b). Enzymatic study on atccd4 and atccd7 and their potential to form acyclic regulatory metabolites. *Journal of Experimental Botany*, 67(21):5993–6005.

Chen, W. W., Schoeberl, B., Jasper, P. J., Niepel, M., Nielsen, U. B., Lauffenburger, D. A., and Sorger, P. K. (2009). Input–output behavior of ErbB signaling pathways as revealed by a mass action model trained against dynamic data. *Mol. Syst. Biol.*, 5(1):239.

Chis, O.-T., Villaverde, A. F., Banga, J. R., and Balsa-Canto, E. (2016). On the relationship between sloppiness and identifiability. *Mathematical Biosciences*, 282:147 – 161.

Coleman, T. F. and Li, Y. (1996). An interior trust region approach for nonlinear minimization subject to bounds. *SIAM J. Opti.*, 6(2):418–445.

Crauste, F., Mafille, J., Boucinha, L., Djebali, S., Gandrillon, O., Marvel, J., and Arpin, C. (2017). Identification of nascent memory CD8 T cells and modeling of their ontogeny. *Cell Systems*, 4(3):306–317.

Fiedler, A., Raeth, S., Theis, F. J., Hausser, A., and Hasenauer, J. (2016). Tailored parameter optimization methods for ordinary differential equation models with steady-state constraints. *BMC Syst. Biol.*, 10(1):80.

Fröhlich, F., Theis, F. J., Rdler, J. O., and Hasenauer, J. (2017). Parameter estimation for dynamical systems with discrete events and logical operations. *Bioinformatics*, 33(7):1049–1056.

Fujita, K. A., Toyoshima, Y., Uda, S., Ozaki, Y.-i., Kubota, H., and Kuroda, S. (2010). Decoupling of receptor and downstream signals in the Akt pathway by its low-pass filter characteristics. *Sci. Signal.*, 3(132):ra56–ra56.

Gutenkunst, R. N., Waterfall, J. J., Casey, F. P., Brown, K. S., Myers, C. R., and Sethna, J. P. (2007). Universally sloppy parameter sensitivities in systems biology models. *PLOS Computational Biology*, 3(10):1–8.

Hass, H., Kipkeew, F., Gauhar, A., Bouch, E., May, P., Timmer, J., and Bock, H. H. (2017). Mathematical model of early Reelin-induced Src family kinase-mediated signaling. *PLoS ONE*, 12(10):1–16.

Hindmarsh, A. C., Brown, P. N., Grant, K. E., Lee, S. L., Serban, R., Shumaker, D. E., and Woodward, C. S. (2005). SUNDIALS: Suite of Nonlinear and Differential/Algebraic Equation Solvers. *ACM T. Math. Software.*, 31(3):363–396.

Isensee, J., Kaufholz, M., Knape, M. J., Hasenauer, J., Hammerich, H., Gonczarowska-Jorge, H., Zahedi, R. P., Schwede, F., Herberg, F. W., and Hucho, T. (2018). Pka-rii subunit phosphorylation precedes activation by camp and regulates activity termination. *J. Cell Biol.*, 217(6):2167–2184.

Kreutz, C. (2018). An easy and efficient approach for testing identifiability. *Bioinformatics*, 34(11):1913–1921.

Kreutz, C. and Timmer, J. (2013). *Optimal Experiment Design, Fisher Information*, pages 1576–1579. Springer New York, New York, NY.

Le Novere, N., Bornstein, B., Broicher, A., Courtot, M., Donizelli, M., Dharuri, H., Li, L., Sauro, H., Schilstra, M., Shapiro, B., Snoep, J. L., and Hucka, M. (2006). Biomodels database: a free, centralized database of curated, published, quantitative kinetic models of biochemical and cellular systems. *Nucleic Acids Res..*, 34:D689–D691.

Leis, J. R. and Kramer, M. A. (1988). The simultaneous solution and sensitivity analysis of systems described by ordinary differential equations. *ACM Transactions on Mathematical Software (TOMS)*, 14(1):45–60.

Lucarelli, P., Schilling, M., Kreutz, C., Vlasov, A., Boehm, M. E., Iwamoto, N., Steiert, B., Lattermann, S., Wäsch, M., Stepath, M., Matter, M. S., Heikenwälder, M., Hoffmann, K., Deharde, D., Damm, G., Seehofer, D., Muciek, M., Gretz, N., Lehmann, W. D., Timmer, J., and Klingmüller, U. (2018). Resolving the combinatorial complexity of smad protein complex formation and its link to gene expression. *Cell Systems*, 6(1):75 – 89.e11.

Merkle, R., Steiert, B., Salopiata, F., Depner, S., Raue, A., Iwamoto, N., Schelker, M., Hass, H., Wäsch, M., Boehm, M. E., Mücke, O., Lipka, D. B., Plass, C., Lehmann, W. D., Kreutz, C., Timmer, J., Schilling, M., and Klingmueller, U. (2016). Identification of cell type-specific differences in erythropoietin receptor signaling in primary erythroid and lung cancer cells. *PLoS Comput. Biol.*, 12(8):e1005049.

Raia, V., Schilling, M., Böhm, M., Hahn, B., Kowarsch, A., Raue, A., Sticht, C., Bohl, S., Saile, M., Möller, P., Gretz, N., Timmer, J., Theis, F., Lehmann, W.-D., Lichter, P., and Klingmüller, U. (2011). Dynamic mathematical modeling of IL13-induced signaling in hodgkin and primary mediastinal B-cell lymphoma allows prediction of therapeutic targets. *Cancer Res.*, 71(3):693–704.

Raue, A., Kreutz, C., Maiwald, T., Bachmann, J., Schilling, M., Klingmüller, U., and Timmer, J. (2009). Structural and practical identifiability analysis of partially observed dynamical models by exploiting the profile likelihood. *Bioinformatics*, 25(25):1923–1929.

Scheff, J. D., Calvano, S. E., and Androulakis, I. P. (2013). Predicting critical transitions in a model of systemic inflammation. *Journal of Theoretical Biology*, 338:9 – 15.

Schelker, M., Raue, A., Timmer, J., and Kreutz, C. (2012). Comprehensive estimation of input signals and dynamics in biochemical reaction networks. *Bioinformatics*, 28(18):i529–i534.

Schwen, L., Schenk, A., Kreutz, C., Timmer, J., Rodriguez, M. B., Kuepfer, L., and Preusser, T. (2015). Representative sinusoids for hepatic four-scale pharmacokinetics simulations. *Plos One*, 10:e0133653.

Sobotta, S., Raue, A., Huang, X., Vanlier, J., Jünger, A., Bohl, S., Albrecht, U., Hahnel, M. J., Wolf, S., Mueller, N. S., et al. (2017). Model based targeting of IL-6-induced inflammatory responses in cultured primary hepatocytes to improve application of the JAK inhibitor ruxolitinib. *Front Physiol.*, 8:775.

Swameye, I., Müller, T., Timmer, J., Sandra, O., and Klingmüller, U. (2003). Identification of nucleocytoplasmic cycling as a remote sensor in cellular signaling by data-based modeling. *Proc. Natl. Acad. Sci.*, 100(3):1028–1033.

Tönsing, C., Timmer, J., and Kreutz, C. (2014). Cause and cure of sloppiness in ordinary differential equation models. *Phys. Rev. E*, 90:023303.

Transtrum, M. K., Machta, B. B., and Sethna, J. P. (2010). Why are nonlinear fits to data so challenging? *Phys. Rev. Lett.*, 104:060201.

Villaverde, A. F., Fröhlich, F., Weindl, D., Hasenauer, J., and Banga, J. R. (2018). Benchmarking optimization methods for parameter estimation in large kinetic models. *Bioinformatics*, bty736.

Waterfall, J. J., Casey, F. P., Gutenkunst, R. N., Brown, K. S., Myers, C. R., Brouwer, P. W., Elser, V., and Sethna, J. P. (2006). Sloppy-model universality class and the vandermonde matrix. *Phys. Rev. Lett.*, 97:150601.

Weber, P., Hornjik, M., Olayioye, M. A., Hausser, A., and Radde, N. E. (2015). A computational model of pkd and cert interactions at the trans-Golgi network of mammalian cells. *BMC Syst. Biol.*, 9(1):9.

Zheng, Y., Sweet, S. M. M., Popovic, R., Martinez-Garcia, E., Tipton, J. D., Thomas, P. M., Licht, J. D., and Kelleher, N. L. (2012). Total kinetic analysis reveals how combinatorial methylation patterns are established on lysines 27 and 36 of histone H3. *Proc. Natl. Acad. Sci. U S A*, 109(34):13549–13554.
